# Chinese people's experience of cancer in the UK: a reflexive thematic analysis

**DOI:** 10.3389/fpsyg.2026.1745349

**Published:** 2026-04-16

**Authors:** Che Ling Michelle Mok, Ho Yan Lai, Amanda C. de C. Williams

**Affiliations:** 1 Research Department of Clinical, Educational & Health Psychology, University College London, London, United Kingdom; 2 Department of Psychology, The University of Hong Kong, Hong Kong, Hong Kong SAR, China

**Keywords:** acculturation, cultural barriers, culture, healthcare access, minority health, psycho-oncology

## Abstract

**Aims:**

This study explored how Hong Kong Chinese cancer patients in the UK perceived their cancer experiences, and how aspects of Chinese culture influenced their access to and engagement with UK health and psychosocial services.

**Methods:**

We conducted reflexive thematic analysis on 10 semi-structured interviews with Hong Kong Chinese individuals who had cancer during the COVID-19 pandemic.

**Results:**

Participants viewed cancer as a continuum that began with an overwhelming sense of death-related fear and worry, followed by physical and mental exhaustion, and frustration over the need to proactively seek help. Communication challenges due to language barriers and cultural differences hindered trust in professionals. However, support from family, friends, and spirituality aided adjustment to cancer.

**Conclusions:**

This study highlights the cancer experiences of a small sample of Hong Kong Chinese people in the UK during the COVID-19 pandemic. Healthcare professionals should be aware of the unique cultural and language-related barriers faced by Hong Kong Chinese cancer patients in the UK. Culturally sensitive communication strategies and accessible practical support are essential to build trust and facilitate engagement with health and psychosocial services. Involving family, community, and spiritual resources in care planning may further support the wellbeing and adjustment of these patients.

## Introduction

The coronavirus SARS-CoV-2 (COVID-19) pandemic had a profound impact on UK cancer services (Archer et al., 2020; Conway et al., 2014; Raymond et al., 2020). Major disruptions of care included suspension of screening, reduced surgical capacity, interrupted or delayed treatment, and face-to-face consultations canceled or replaced by online appointments (El-Shakankery et al., 2020; Jones et al., 2020; Spicer et al., 2020). These adversely affected cancer care experiences, and increased mortality rates (Archer et al., 2020; Hanna et al., 2020; Jones et al., 2020; Office for Health Improvement and Disparities, 2023; Richards et al., 2020). Cancer patients were advised in addition to avoid social contact, for prolonged periods, at a time of increased stress. This, combined with limited availability of psychosocial support during the pandemic, further limited cancer patients' support to mitigate distress and loneliness (Seven et al., 2021).

Although telemedicine offered a viable alternative during the pandemic (Eberly et al., 2020; Haleem et al., 2021; Jin et al., 2020; Zhu et al., 2022), the rapid transition in the UK from face-to-face consultations to online appointments exacerbated existing cancer inequalities (Abraham et al., 2022). Socioeconomically disadvantaged patients often lacked the resources for telemedicine (Eberly et al., 2020; Ramirez et al., 2021; Zhu et al., 2022), and cancer patients from ethnic minorities faced numerous barriers, including language and communication gaps, financial constraints, inadequate healthcare services, and poor understanding by healthcare providers of their religious, cultural, and spiritual beliefs about illness, gender roles, and family obligations (Elkan et al., 2007; Tam-Ashing et al., 2003). Non-English-speaking ethnic minorities reported more negative care experiences and struggled with lower health literacy than English-speaking ethnic minorities, leading to lower treatment and perceived poorer healthcare (Kagawa-Singer et al., 2010; Holden et al., 2021; Raleigh and Holmes, 2021).

At least one third of the Hong Kong Chinese population in the UK lives in London (Office for National Statistics, 2021), with a substantial increase in immigration since January 2021 under a new visa (Donaldson, 2021; Office for National Statistics, 2022). The demographic profile of the Hong Kong Chinese population is diverse, including British-born Chinese people, students from mainland China and Hong Kong, and Hong Kong-born-and-raised adults and their dependants who are generally older, less fluent in English, and less familiar with British culture (Chan et al., 2007; Office for National Statistics, 2021).

The Chinese community in the UK is often viewed by UK statutory bodies as silent and self-sufficient, relying on family and community support rather than on statutory sources (Chan et al., 2007; National Children's Centre, 1982; Runnymede Trust, 1986). This stereotype, based on limited knowledge of the Chinese community, disadvantages Chinese people in the UK (Chau, 2008; Lam, 2002). Among Chinese people, older people, women, and middle-aged men working in family catering businesses were more likely to be excluded from UK health services because of language barriers, social isolation, and difficulties accessing services (Tran, 2006; Yu, 2000). Factors such as traditional health beliefs, family roles, and settlement patterns influence their use of UK health services, yet many Western healthcare professionals struggle to integrate this knowledge into practice (Chau and Yu, 2002, 2004; Gervais and Jovchelovitch, 1998; Healthcare Commission, 2008).

Only one qualitative study has explored the meaning of cancer for Chinese people living in London, with family members or close friends of Chinese descent with cancer (Papadopoulos et al., 2007). The study highlighted common beliefs about cancer causation and concerns about disclosure and stigma, consistent with findings from Chinese cancer patients in the US and Australia (Gonzalez et al., 2015; Huang et al., 2008; Kwok and White, 2011; Lui et al., 2009; Papadopoulos et al., 2007; Tam-Ashing et al., 2003; Wong-Kim et al., 2005).

The present study, therefore, aimed to (1) describe how Hong Kong Chinese cancer patients in the UK experience cancer and cancer healthcare; and (2) identify aspects of Chinese culture that may influence their access to and engagement with UK health and psychosocial services.

## Method

### Design and setting

This qualitative reflexive thematic analysis study had ethical approval from the UCL Research Ethics Committee (reference 23527/001). Participants were recruited through study posters in English, traditional Chinese, and simplified Chinese, distributed via Cancer Research UK's online newsletter, notice boards at Maggie's London cancer centers (www.maggies.org), the Chinese Association for Cancer Care's WhatsApp support group, and the first author's social media platforms, including Facebook, Instagram, and X (formerly Twitter), between November 2022 and February 2023.

### Participants

Eligible participants were at least 18 years old and identified as Asian Chinese, and agreed to a video- and audio-recorded, individual, approximately hour-long semi-structured interview conducted by the first author on Microsoft Teams. Those receiving palliative care services were excluded. Eleven participants expressed interest, met criteria, and consented to participation. One participant withdrew before the interview due to departure from the UK.

### Sample size and information power

Data saturation, the point at which no new codes or themes emerge from the data, is widely used to justify sample sizes in qualitative research. However, Braun and Clarke (2021) have argued that this concept is epistemologically incompatible with reflexive thematic analysis, since themes are not “discovered” in the data, but actively generated through the researcher's interpretive engagement.

Rather than considering saturation, we drew the concept of “information power” (Malterud et al., 2016) to justify our sample size. This holds that the more relevant information a sample provides related to the study's aim, the fewer participants are needed. Here information power was strengthened by (a) a clearly defined and narrow aim for the study—understanding the experiences of Hong Kong Chinese cancer patients in the UK during the COVID-19 pandemic; (b) drawing participants from a specific, culturally homogeneous population directly relevant to the research question; (c) in-depth interviews conducted in participants' first language (Cantonese), facilitating rich and detailed accounts; and (d) analysis by reflexive thematic analysis with both experiential and critical orientations well-suited to generating meaningful interpretations.

### Procedure

Interested potential participants were directed to more information about the study, and to an online form on which to register their interest by completing questions on the inclusion criteria and contact details. When the researcher contacted them, she answered any questions, requested return of the consent form, and arranged an interview online. Instruction on the use of Microsoft Teams for the interview was provided where necessary.

### Semi-structured Interviews

Interviews followed a semi-structured protocol in English developed and revised by the first author with two psycho-oncology colleagues and reviewed by an expert by experience who is the founder of the Chinese Association for Cancer Care (Appendix A). The protocol was translated into traditional and simplified Chinese by the first author (a native Chinese speaker), and addressed two key areas: (1) understanding cancer diagnosis and living with and beyond cancer in the UK; and (2) experience of accessing and engaging with the UK health and social care system and psychosocial support during the COVID-19 pandemic.

All interviews were conducted in Cantonese and transcribed verbatim by the first author. Translation into English was also undertaken by the first author, who is a native Cantonese speaker fluent in English. We recognize that translation is not a neutral process but an interpretive act that shapes the data available for analysis (Temple and Young, 2004; Squires, 2009). Translation choices were discussed during supervisory dialogue with AW, particularly where expressions carried cultural meanings that risked being flattened or pathologised in English rendering. We prioritized meaning equivalence over literal translation, consistent with recommendations for cross-language qualitative research (van Nes et al., 2010).

### Data analysis

#### Qualitative frameworks

The first part of the study aimed for an experiential account of participants' experiences of living with and beyond cancer in the UK; the second part employed more critical analytic methods to link findings to Chinese culture and ideologies (Lawless and Chen, 2019).

#### Ontological and epistemological positions of the researcher

The first author-researcher's critical realist ontology and constructivist epistemology guided methodological choices (Creswell, 2009; Edwards et al., 2014; Hesse-Biber, 2016; Fryer, 2022; Given, 2008; Patton, 1990; Taylor, 2018). An inductive, bottom-up approach was used to develop themes and sub-themes, coding data without reference to pre-existing frameworks or the researcher's analytic preconceptions (Braun and Clarke, 2006; Gale et al., 2013). This allowed exploration of underlying ideas and assumptions shaping the data (Braun and Clarke, 2006), acknowledging that Chinese cultural influences on an individual's belief and behavior might not be consciously recognized by participants (Jovchelovitch and Gervais, 1999; Tebes, 2005).

### Analytic procedure

Transcribed interviews were analyzed using Nvivo software, following the six iterative phases of reflexive thematic analysis (Braun and Clarke, 2006, 2022a,b). The first author transcribed interviews verbatim, anonymising and translating from Cantonese to English. Repeated readings and video reviews facilitated deep familiarization with the data and generation of initial descriptive codes. Codes were collated into candidate themes, which were iteratively conceptualized and refined, moving back and forth between the full dataset, coded extracts, and developing themes, to form more analytically salient themes. This recursive process, which (Braun and Clarke 2022a,b) and Terry and Hayfield (2020) identify as central to rigor in reflexive thematic analysis, ensured that themes were continually tested and refined against data (Forero et al., 2018; Roberts et al., 2019). The sequence was repeated for each interview before clustering themes across all interviews to identify overarching patterns. An audit trail of evolving codes, candidate themes, and thematic maps was maintained throughout to support dependability and confirmability (Lincoln and Guba, 1985). Thematic maps were used to visualize conceptual relationships between themes and sub-themes for each research question.

### Positionality and trustworthiness

The first author was a trainee clinical psychologist completing her doctoral research at University College London. She had MSc degrees in Mental Health Studies from King's College London and in Psychology of Mental Health from University of Edinburgh. This was her first research in psycho-oncology, but she had prior experience in qualitative thematic analysis (Mok et al., 2020). She is committed to improving the healthcare experiences of immigrants and ethnic minority service users in the UK. As a Hong Kong Chinese female raised in Hong Kong and acculturated to the UK, and also as a trainee clinical psychologist having worked with cancer patients in London, she was mindful of her experiences informing results. Rigor was maintained through supervisory dialogue with the last author (AW), a form of “critical friendship” (Anderson, 2010; Smith and McGannon, 2018) distinct from inter-rater reliability checking, as the latter can be considered incompatible with reflexive thematic analysis (Braun and Clarke, 2022a,b). These discussions encompassed method alignment, critical interrogation of developing themes and their conceptualization, review of thematic maps, and reflexive examination of how the first author's positionality and assumptions shaped the analysis (Attride-Stirling, 2001). AW's unfamiliarity with Chinese culture provided an analytic counterpoint, prompting the first author to make explicit her interpretations (Braun and Clarke, 2006; Carter et al., 2014; Denny and Weckesser, 2019; Leung, 2015). The first author maintained a reflexive journal throughout data collection and analysis, documenting personal reflections, assumptions, emotional responses to the data, and evolving interpretive decisions. This practice is consistent with the centrality of researcher reflexivity within Braun and Clarke's (2019, 2022a,b) approach, in which the researcher's subjectivity is understood not as problematic bias but as a resource shaping the analytic process. A COREQ checklist is provided (Appendix B). AW is an academic and clinical psychologist, with over 35 years' experience working in chronic pain, including with cancer survivors, but she is unfamiliar with Chinese culture in relation to healthcare. The second author, HYL, is a research assistant with a Bachelor of Psychology from The University of Hong Kong, who was not involved in interviews and data analysis.

Consistent with Lincoln and Guba's (1985) framework, several strategies enhance the trustworthiness of this study. Credibility was supported through in-depth engagement with interviewees, the reflexive journal, and regular supervisory dialogue (detailed above). Dependability and confirmability were supported by maintenance of a detailed audit trail and transparent reporting of analytic decisions. Transferability is facilitated through the provision of thick description of participants' backgrounds, the interview context, and the sociocultural setting, enabling readers to assess the applicability of findings to other contexts (Lincoln and Guba, 1985). In keeping with the epistemological orientation of reflexive thematic analysis, we invite readers to make their own judgements about whether and how these findings resonate with other populations or settings.

## Results

### Participant information

Ten participants with varied cancer diagnoses who identified themselves as Hong Kong Chinese people living in England and Northern Ireland, aged between 39 and 68, were interviewed on Microsoft Teams, in their own homes with nobody else present. All were recruited from the Chinese Association for Cancer Care and opted to be interviewed in Cantonese Chinese (see Table 1 for participants' demographic information and treatments). There was no technological failure or problem in recording and transcribing.

**Table 1:** Participants' demographic information.

ID	Sex	Age range	Marital status	No. of children	Employment status	Duration of living in the UK (years)	On BNO visa?	Year of cancer diagnosis	Type of primary cancer diagnosis
1	F	36–40	Married	1	Unemployed	5–10	No	2022	Breast
2	F	51–55	Single	0	Unemployed	Under 5	Yes	2021	Breast
3	F	56–60	Married	1	Unemployed	Under 5	Yes	2021	Breast
4	F	41–45	Single	0	Working part-time	Under 5	Yes	2021	Lung
5	F	41–45	Single	0	Working part-time	More than 10	No	2021	Breast
6	F	41–45	Married	1	Graded return to work	More than 10	No	2021	Breast
7	M	51–55	Married	2	Self-employed	More than 10	No	2020	Esophageal
8	F	66–70	Married	2	Unemployed	More than 10	No	2020	Breast
9	F	36–40	Single	0	Graded return to work	Under 5	Yes	2021	Breast
10	M	51–55	Married	1	Unemployed	Under 5	Yes	2021	Colorectal

Six themes were identified for the Research Question 1, narratively organized to reflect participants' understanding of their cancer experience, and three themes for Research Question 2, arranged by thematic salience. Chinese idioms and colloquialisms are provided in brackets to preserve cultural and linguistic nuances. All themes are illustrated with verbatim extracts and two conceptual models.

### Research question 1: how was cancer experienced by Hong Kong Chinese people in the UK during the COVID-19 pandemic?

#### Theme 1: an overwhelming sense of death-related fear and worry

Participants reported that receiving a cancer diagnosis triggered intense fear and worry about death.

##### Sub-theme 1.1: i have lost control of my life

In recalling their thoughts and emotions on receiving their cancer diagnosis, participants described cancer as “*uncontrollable*” and “*smothering*”; a “*death sentence*,” which triggered “*counting down of days*.” The diagnosis of cancer left them “*shocked*,” “*surprised*,” “*numb*,” and “*scared*” because of the death-related fear and uncertainty about life with cancer.

“*I felt bewildered* (六神無主) *and immediately thought about my impending death and the list of things that I needed to do before dying, such as choosing a photograph for my funeral*.” (Participant 3, female, breast cancer)

##### Sub-theme 1.2: cancer stripped my ability to take care of my family

Post-diagnosis, participants described feeling overwhelmed by the anxiety-laden, “what-if” thoughts about failing to take care of their families due to illness or death.

“…*my son is still very young. He needs me to be there for him, but what if I can't?*” (Participant 1, female, breast cancer)

Family care responsibilities felt paramount, and cancer “*robbed*” people of their ability to discharge these duties, causing sadness, disappointment, guilt, and remorse. Some felt anguish at becoming the one needing care, lamenting lost opportunities to be a “*good son*” or “*good daughter*.”

“*My plan was to invite my parents from Hong Kong to come over and live with me after I settled down. But now cancer has completely ruined my plan. Who can take care of my parents?*” (Participant 2, female, breast cancer)

“*I don't usually cry. But when I knew I had cancer, I cried. What if I die before my parents do?*” (Participant 7, male, esophageal cancer)

#### Theme 2: cancer has been physically and emotionally exhausting

This theme concerns participants' prolonged experience of the physical, emotional, and social impacts of cancer. For some, the physical pain of cancer, described as “*excruciating*,” “*agonizing*,” and “*paralyzing*,” and the side effects of chemotherapy, were at the core of this experience; others felt exhausted from enduring pain, with daily pain robbing them of joy (食唔安坐唔 樂).

“*If surviving means having to suffer from pain every day without being able to do much, is it still meaningful to stay alive?*” (Participant 2, female, breast cancer)

Younger female breast cancer patients struggled with treatment-induced long-term changes to appearance and physical function, and reflected that they had underestimated the emotional and social impact of losing breasts or fertility.

“*I found it very difficult to take a shower or look at myself in the mirror to see that I no longer have my nipples on my reconstructed breasts. It was very hard to talk to others about it*.” (Participant 9, female, breast cancer)

Some felt outraged that mastectomy and chemotherapy were presented as “*the best*” treatment options with little attention to their psychological impact; this concern is further explored under Research Question 2 in relation to cultural expectations of the clinical relationship.

“*I would have appreciated it if someone sat down and went through with me slowly what all the changes meant to me*.” (Participant 5, female, breast cancer)

Several participants were exhausted from trying to remain hopeful, and felt alienated from healthy individuals and the dominant narrative of focussing on the positives.

“*Every part of my life has been affected by cancer…. People sometimes encourage me to think about the positives, but the reality is that cancer is smothering my life*.” (Participant 4, female, lung cancer)

#### Theme 3: frustration about having to be proactive in seeking help in the UK

Participants expressed frustration at needing to be proactive in their NHS care despite their physically and emotionally vulnerable state. They were exasperated by medical errors and desired more definitescheduling of follow-up appointments, up-to-date and accurate patient records, and healthcare professionals being more available for support.

“*I thought as a patient I did not have to worry about stuff such as making sure my patient record was up to date, appointments were booked in, and blood tests were in place. But in reality, I had to do all of these because there were so many errors and it was extremely stressful*.” (Participant 1, female, breast cancer)

Newly migrated participants diagnosed with cancer shortly after arrival in the UK struggled with adjustment to a different healthcare system and unfamiliarity with UK conventions.

“*I was used to getting my prescribed medicine directly at the clinic where I saw the doctor. But here in the UK I needed to go to a pharmacy. Nobody told me where I should go!*” (Participant 10, male, colorectal cancer)

#### Theme 4: lack of trust in UK health and psychosocial care professionals

Participants described their lack of trust in healthcare professionals as “*irreversible*,” compounding the exhaustion and frustration described above. Five sub-themes highlight contributing factors. While one participant sought private healthcare through work insurance, most lacked the resources to do so, and found NHS care lacked the transparency, consistency, and cultural sensitivity they needed.

##### Sub-theme 4.1: language barrier

Participants described a “*wall*” between themselves and healthcare staff. Those who spoke English were uncomfortable articulating feelings in English, undermining possibilities of using cancer psychosocial support.

“*I am comfortable in speaking in English at work and in casual daily conversations. But I didn't feel comfortable talking about how I really felt, especially about cancer in English*.” (Participant 9, female, breast cancer)

Those using NHS interpreter services often had poor experiences of inconsistent quality and feeling “*silenced*.”

“*Interpreters in the NHS never do more than the bare minimum. They often made me feel they were in a rush*.” (Participant 8, female, breast cancer)

##### Sub-theme 4.2: cultural differences between UK and Hong Kong on family, healthcare, and recovery

Fundamental differences between UK and Hong Kong cultures in relation to family, healthcare, and recovery made participants feel disconnected when communicating with professionals.

“*I was encouraged by my therapist to go out and meet new friends to heal my home-sickness of missing my family. But hearing that just made me feel she didn't understand how different family is to me compared to other people*.” (Participant 2, female, breast cancer)

Some were perplexed by oncologists asking their treatment preferences, a conflict of cultural expectations explored further under Research Question 2.

“*I instantly thought how would I know? He was supposed to be the expert!*” (Participant 10, male, colorectal cancer)

Others desired a more holistic perspective on their cancer experience and recovery.

“…* conversations [with NHS staff] often felt less personal, less warm, and more like an interview than when I talked to volunteers from the Chinese Association for Cancer Care… When I was with other Chinese cancer patients and volunteers from the Chinese Association for Cancer Care, we talked more about how everything we were going through was just a natural part of the journey called life. That made me feel more comfortable and connected to other people*.” (Participant 6, female, breast cancer)

##### Sub-theme 4.3: lack of consistency in oncology consultants

Distrust in professionals was associated with encountering different oncology specialists each time, which felt “*strange*” and “*bewildering*,” and trust was entirely lost if they received “*conflicting advice*” from different oncologists.

“*There was no continuity in consultants* – *different consultant each time… Sometimes consultants even gave me conflicting advice: e.g., one consultant advised me to go out for walks more often whilst another advised me to stay indoors and go out less. Who should I trust?*” (Participant 10, male, colorectal cancer)

##### Sub-theme 4.4: frequent unexplained appointment cancellations and unavailability of Clinical Nurse Specialists (CNS)

Lost trust in the NHS was also attributed to frequent, last-minute unexplained cancellations of appointments. Some wondered whether COVID-19 had depleted resources and staff, accounting for the problem.

“*I felt stressed each time before I had to go to the hospital for my chemotherapy. So finding out that my chemo was actually cancelled or booked on the wrong date when I made it to the hospital was very frustrating*.” (Participant 5, female, breast cancer)

##### Sub-theme 4.5: professionals' lack of understanding of the BNO visa status

Recent immigrants felt alienated due to professionals' lack of knowledge about their visa conditions, fuelling participants' sense of disconnection and powerlessness, frustration and skepticism about available support.

“*Services spent a lot of time on assessing my needs. But in the end, they often said I needed to be referred onwards because they found out that they were not funded to support BNO visa holders without permanent citizenship. I felt frustrated because the assessments were a waste of my time and energy*.” (Participant 2, female, breast cancer)

#### Theme 5: cancer suffering was alleviated by support to manage practical challenges, spirituality, and kind words from people I trusted

Participants identified support with practical matters, such as “*traveling to hospital appointments*,” “*cooking*,” “*helping with household chores*” and “*calling services*” on their behalf as helpful, mitigating suffering associated with cancer, and easing their difficulties in “*some of the hardest times*.” Participants valued what their family and friends voluntarily did for them after diagnosis.

“*My husband decided to buy a car [after hearing about my cancer diagnosis] to take me to hospital appointments. That made me feel we were a team*.” (Participant 3, female, breast cancer)

Participants also described that they felt “*accompanied*” and “*taken care of* ” by volunteers from a Chinese community organization who offered help navigating the health and social systems in the UK.

“*The volunteer I had from the Chinese Association for Cancer Care was fantastic, as she was not only my interpreter in my doctors' appointments, but she also helped me liaise with local rehabilitation services to make sure I knew where to look for support*.” (Participant 8, female, breast cancer)

In addition to the practical day-to-day support, spirituality played an extremely important role as a life compass, offered direction and peace, while kind words from trusted individuals provided comfort.

“*Spirituality and religion gave me a ‘virtual figure' that I could talk to, where I could gain some inner peace from when life became too overwhelming*.” (Participant 1, female, breast cancer)

“*I felt deeply cared for when a friend reminded me of how much I meant to her and how she would be very sad if she lost me*.” (Participant 4, female, lung cancer)

#### Theme 6: radical acceptance of the suffering associated with cancer

This theme reflects the concept of radical acceptance (Linehan, 1993): accepting reality for what it is, even if far from ideal. After registering the impact of cancer, some participants found a way to be self-compassionate, with tenderness and humor in tough times.

“*I had always complained about having too much hair before, so now that I am bald, I will never have any bad hair day! I also told my husband that ‘you are bald anyway, so why is it a problem if I am bald?'*” (Participant 6, female, breast cancer)

Some described personal growth through their cancer experience, explaining it using Chinese proverbs learned as a child.

“*As the old Chinese saying goes, ‘you need to lose something to gain something'* (有得必有失).” (Participant 3, female, breast cancer)

**Figure 1:**
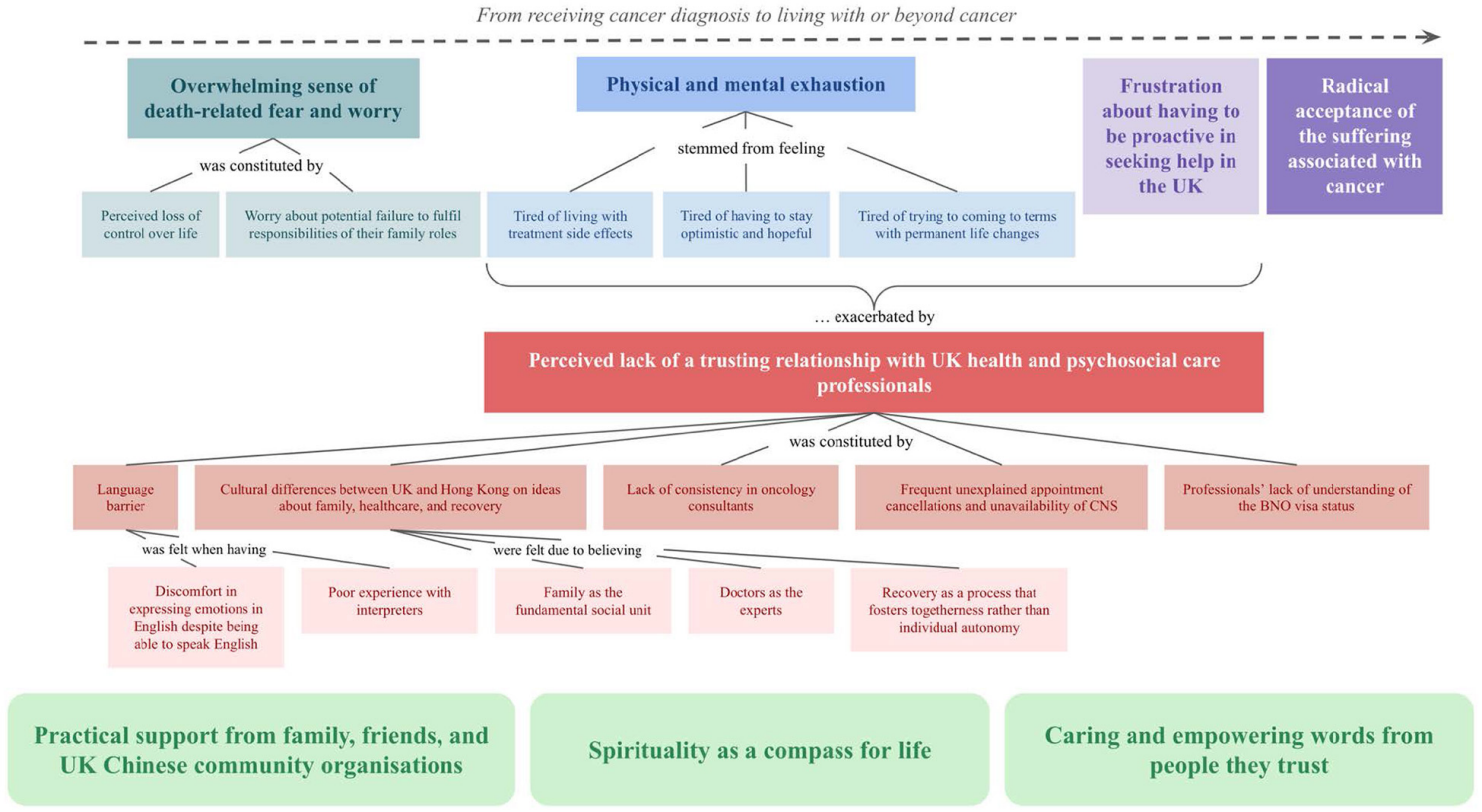
Cancer experiences of Hong Kong Chinese people in the UK.

See Figure 1 for the thematic map of the themes and sub-themes.

### Research question 2: what aspects of Chinese culture affect Hong Kong Chinese cancer patients' access to and engagement with UK health and psychosocial services?

#### Theme 1: challenging the doctor's opinion violates my cultural values of respect and politeness

Most participants believed that healthcare professionals, especially doctors, were experts whose opinions “*should not be challenged*,” even when participants doubted them, because of the power dynamics in patient-oncologist relationships and the important Chinese cultural value of humility, making questioning seem “*disrespectful*.”

“*Although I don't always agree with my consultants, at the end of the day, they are still the doctors*.” (Participant 2, female, breast cancer)

“*If I challenge my doctors, I feel like I am being arrogant. I need to be humble*.” (Participant 8, female, breast cancer)

This deference led participants to suppress stating their treatment preferences and concerns,. so when doctors' recommendations subsequently proved incomplete or inaccurate, participants felt betrayed. The very deference that prevented them from questioning became a source of broken trust.

“*Doctors have more experience than I do, so I should trust them despite my concerns about mastectomy*.” (Participant 5, female, breast cancer)

“*I didn't understand why reconstruction wasn't offered as an option to pursue along with mastectomy before the surgery. Nobody seemed to care about whether I had plans to have kids and whether I wanted to consider egg-freezing before starting on chemotherapy. Now that I will never be able to have kids, I just feel like my doctor encouraged me to do what was best for the service at that time, not what was best for me, because mastectomy without reconstruction was the quickest and simplest option. The fact that I could have kept my breasts and frozen my eggs angers me*.” (Participant 5, female, breast cancer)

Participants' compliance with doctors' advice and reluctance to raise concerns were apparently misinterpreted by Western clinicians as an absence of concerns or of distress, leading to missed opportunities to rebuild trust.

#### Theme 2: asking for more than what I am offered is inconsiderate to others

This theme reflects the impact of collectivist culture on participants' engagement with services.

“*Western culture expects me to ask for help; but I am not used to asking for help, I am used to receiving what is offered*.” (Participant 2, female, breast cancer)

Participants felt they needed to be assertive to obtain support in the UK, but this conflicted with cultural norms.

“*Asking for more than what I am offered makes me feel I am creating additional workload for others, and I don't want to bother others*.” (Participant 5, female, breast cancer)

Participants' reluctance to ask for help from a desire not to burden others is related to a fundamental component in Chinese cultural collectivism, where individual needs are secondary to those of the group. One participant described her appreciation for peer support in the Chinese community organization for cancer.

“*Chinese people don't like to ask for help. We try to be supportive to each other in action before we even have to ask for support*.” (Participant 6, female, breast cancer)

#### Theme 3: i am accustomed to enduring pain without showing much emotion

Older participants were more likely than their younger counterparts to feel that psychological support was “*unnecessary*” when coping with cancer distress.

“*I don't think people need to talk about the negative things so much*.” (Participant 8, female, breast cancer)

Men were more likely than women participants to articulate their belief in the idea of karma, referencing the old Chinese saying which says, “*if you eat a salty fish, then you should expect to be thirsty after*” (食得鹹魚 抵得渴).

“*I did not contact any psychological or counselling support service, as I knew I had only got myself to blame for my cancer. I did so many wrong things in the past*.” (Participant 10, male, colorectal cancer)

Older and particularly male participants, influenced by values of enduring pain without showing emotion, generally felt no need for psychological support, preferring practical help.

“*I feel the UK system often makes you talk and feel like there are a lot of different services to support your different needs. But when you look closer, what they often do is just talking using comforting words that make no actual difference to my life, and then signposting me to somewhere else. It is an exhausting process*.” (Participant 2, female, breast cancer)

**Figure 2:**
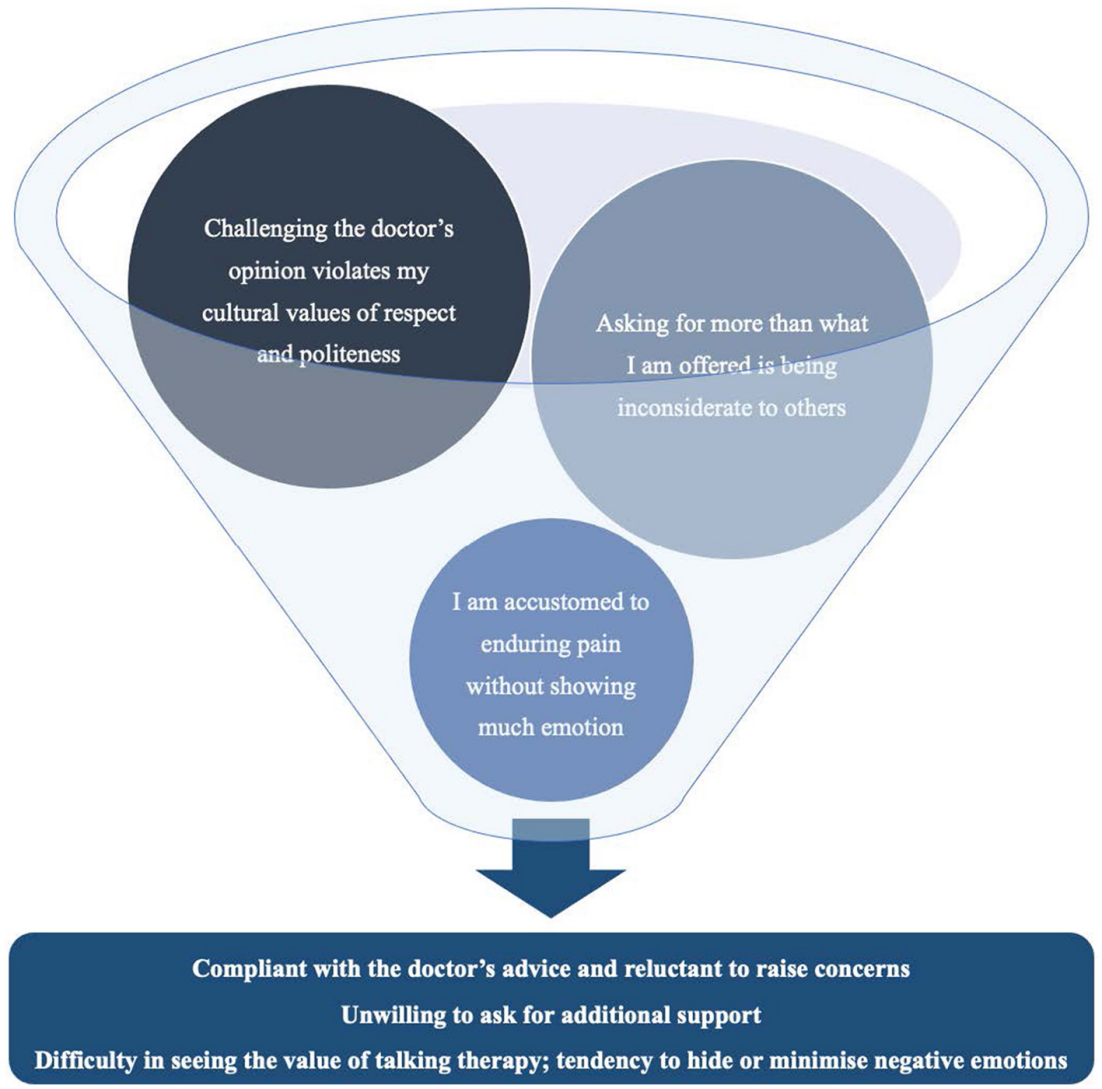
Traditional Chinese cultural ideologies reported to affect Hong Kong Chinese people's access to or engagement with UK health and psychosocial services.

Most participants endorsed at least two of these three themes, particularly the first two, suggesting that these cultural values combined in shaping engagement with services. Notably, stoicism was endorsed predominantly by participants who had lived in the UK for more than a decade, whereas deference to authority and collectivistic help-seeking norms were endorsed more equally across recent and longer-term immigrants. Figure 2 presents a conceptual model of how these ideologies affected Hong Kong Chinese cancer patients' access to and engagement with UK cancer support services. Table 2 is a summary of themes and sub-themes for research questions 1 and 2.

**Table 2:** Summary of themes and sub-themes for research questions 1 and 2.

Research question	Theme	Sub-themes/key components
RQ1: How was cancer experienced by Hong Kong Chinese people in the UK during the COVID-19 pandemic?	1. An overwhelming sense of death-related fear and worry	1.1 I have lost control of my life
		1.2 Cancer stripped my ability to take care of my family
	2. Cancer has been physically and emotionally exhausting	Physical pain and treatment side effects; treatment-induced changes to appearance and function; exhaustion from maintaining hope; alienation from positivity narratives
	3. Frustration about having to be proactive in seeking help in the UK	Burden of managing own NHS care; medical errors and record inaccuracies; unfamiliarity with UK healthcare conventions (newly migrated participants)
	4. Lack of trust in UK health and psychosocial care professionals	4.1 Language barrier
		4.2 Cultural differences between UK and Hong Kong on ideas about family, healthcare, and recovery
		4.3 Lack of consistency in oncology consultants
		4.4 Frequent unexplained appointment cancellations and unavailability of CNS
		4.5 Professionals' lack of understanding of the BNO visa status
	5. Cancer suffering was alleviated by support to manage practical challenges, spirituality, and kind words from people I trusted	Practical support; community organization support; spirituality as life compass; kind words from trusted individuals
	6. Radical acceptance of the suffering associated with cancer	Self-compassion; humor in adversity; personal growth; Chinese proverbs as meaning-making frameworks
RQ2: What aspects of Chinese culture affect Hong Kong Chinese cancer patients' access to and engagement with UK health and psychosocial services?	1. Challenging the doctor's opinion violates my cultural values of respect and politeness	Deference to medical authority → suppressed concerns and treatment preferences → compliance misread as absence of distress → broken trust when recommendations prove incomplete
	2. Asking for more than what I am offered is inconsiderate to others	Collectivist orientation → reluctance to request additional support → unmet needs → disengagement from services
	3. I am accustomed to enduring pain without showing much emotion	Stoicism → rejection of psychological support → preference for practical and directive help → frustration with assessment-heavy psychosocial pathways

## Discussion

This qualitative study explored how Hong Kong Chinese cancer patients in the UK experienced cancer and cancer care during the COVID-19 pandemic, and how elements of Chinese culture shaped their experiences with UK health and psychosocial services. Themes experiences reported by other UK-based cancer patient groups, but revealed culture-specific patterns related to authority, collectivism, and stoicism that importantly influenced communication, help-seeking, and trust.

### Shared cancer experiences

We found numerous parallels between our participants' experiences and those reported in wider UK cancer populations, including perceptions of cancer as a “death sentence,” fatalistic responses, and the framing of treatments as necessary but harmful (Cockle and Ogden, 2022; Ellis et al., 2005; Licqurish et al., 2016; Quaife et al., 2015). Psychosocial challenges commonly described by breast cancer survivors, including negative body image, reduced self-esteem after mastectomy, and treatment-induced infertility, were prominent in our largely breast-cancer sample (Carreira et al., 2021; Koçan and Gürsoy, 2016; Lam and Fielding, 2003; Iyer and Ring, 2017; Rahool et al., 2021). These themes were amplified by immigration-related disadvantages such as socioeconomic precarity, unfamiliar systems, lower health literacy, and limited acculturation (Lee S. et al., 2013; Levesque et al., 2019; Wu et al., 2019). Participants' emphasis on support from families, close friends, and spirituality aligned with existing literature on the family-oriented nature of Chinese society (Hou et al., 2009; Lui et al., 2009; Mok et al., 2010).

### Communication and trust

Participants experienced pervasive language and culturally mediated communication difficulties that undermined trust in clinicians, and those using NHS interpreter services reported feeling rushed and silenced, consistent with other ethnic minority patients' experiences (Rocque and Leanza, 2015). However, English-speaking participants also reported negative experiences characterized by discomfort articulating feelings in English, unfamiliarity with UK consultation structures, and culturally different communication styles. While cross-cultural competency in healthcare has been extensively researched (Crezee and Roat, 2019; Kagawa-Singer et al., 2010), specific guidance for working with Hong Kong Chinese patients in the UK remains limited. Communication difficulties extended beyond language to culturally different assumptions about clinical encounters (Chichirez and Purcărea, 2018; Elkan et al., 2007; Karbani et al., 2011; Pinder et al., 2016; Magadi and Magadi, 2022).

### Culture-specific ideologies

Several findings were specific to Chinese culture. First, participants respected authority in healthcare settings, so clinicans' attempts to reduce the power differential conflicted with patients' cultural frame of reference, so could lead to misinterpretation of Chinese patients' silence as a lack of distress or concern. In many Chinese societies, particularly in Hong Kong, doctors are often revered as heroes who save lives (Ng, 2023; Wang and Du, 2023), and respectful compliance is related to a core value from Confucianism (Chien, 2016). The UK healthcare system's commitment to shared decision-making, with active patient participation in treatment choices (NICE, 2021), assumes a degree of individual autonomy and assertiveness that is at odds with deference to medical authority common among Chinese patients (Hawley and Morris, 2017; Zhai et al., 2020). A family-centered decision-making model involving the patient's role, family functional structure, and information control, differs fundamentally from the dyadic clinician-patient model of NHS practice (Zhai et al., 2020). A systematic review of Asian Americans found that patients navigated competing expectations between their own cultural frameworks and the norms of the host healthcare system (Tan et al., 2022), a tension expressed by our participants.

Second, participants described a reluctance to burden others across multiple contexts of healthcare and family roles. Rooted in Confucian values and collectivist orientations (Bedford and Hwang, 2003; Chan et al., 2007; Lee R. M. et al., 2013; Warmoth et al., 2017; Wu et al., 2019), this resonates with the characterization of the Chinese community in the UK as “silent and self-sufficient” (Chan et al., 2007; Rudat, 1994), as reluctance to request support and self-advocate in services is misread as an absence of need.

Third, stoicism influenced some participants' engagement with services, with older participants more likely to keep cancer news within the family (Chu et al., 2021; Lee and Shi, 2022; Lui et al., 2009). Stoicism and stigma-related non-disclosure were most prominent among older participants (aged 55+) and those who had emigrated from Hong Kong more than a decade ago, possibly reflecting both generational attitudes and the influence of longer exposure to UK norms that may reinforce self-reliance (e.g., the characterization of the Chinese community as “silent and self-sufficient”; Chan et al., 2007). By contrast, younger participants with more recent Hong Kong residence appeared more comfortable discussing cancer openly and engaging with psychosocial services, which may reflect modernization of attitudes in contemporary Hong Kong, including population-based campaigns to reduce cancer stigma (Department of Health, Food and Health Bureau, and Hospital Authority, 2019; So et al., 2022; Wang and Wang, 2019). Deference to authority and collectivist reluctance to burden others, however, were endorsed more broadly across the sample, suggesting that these ideologies may be more resistant to generational or acculturation-related shifts—possibly because they are embedded in everyday relational norms (e.g., filial piety, politeness, face management) rather than disease-specific attitudes. Our sample was predominantly female (8 of 10 participants) and predominantly comprised breast cancer survivors. We are therefore cautious about drawing gender-specific conclusions. However, it is worth noting that the two male participants in our study described their experiences of stoicism and emotional restraint in ways that appeared to intersect with both cultural expectations and masculine norms around emotional expression. Research on Chinese masculinity suggests that men may face a “double bind” of cultural stoicism and gendered expectations of emotional control (Chu et al., 2021; Levant et al., 2003), which could compound reluctance to engage with psychosocial services. This warrants further investigation with a larger and more gender-balanced sample.

### How cultural ideologies intersect with features of NHS cancer care

Our findings are best understood not as the a simple product of culture, but as arising from interaction between culturally shaped expectations and specific features of UK healthcare (Markus and Kitayama, 1991). For participants who valued deference to authority, shared decision making style could be confusing or indicate that clinicians were withholding expertise. For participants oriented toward collectivism and harmony, the need for patients to request support (e.g., chasing referrals, repeatedly explaining needs or concerns) could feel morally uncomfortable, risking appearing demanding or burdensome. Finally, for participants shaped by stoicism, psychosocial pathways that require repeated assessment and disclosure, rather than practical problem-solving, could feel misattuned. All can lead to fatigue and disengagement, difficulty repairing relationships once trust was damaged. Making the rationale for participation explicit, offering structured summaries, and providing culturally safe “permission” to raise concerns may help to bridge these differences.

Two cross-cultural communication frameworks help to organize these findings. Kleinman's (1980) explanatory model highlights patients' and clinicians' different understandings of illness, recovery, and what constitutes a “good” consultation; when these are not explicit, misalignment can appear as non-engagement or poor rapport. For instance, when participants described doctors as uncaring because they invited questions rather than directing treatment, this can be understood through Kleinman's framework as a mismatch between the patient's explanatory model—in which a good doctor demonstrates authority—and the clinician's model, which equates patient participation with good care. A cultural humility lens (Tervalon and Murray-García, 1998) focuses on clinicians' and services' responsibility to recognize blind spots, negotiate meaning, and adapt communication in partnership with patients. For example, a cultural humility approach would prompt clinicians to question whether their interpretation of a patient's silence reflects the patient's actual experience or their own culturally shaped assumptions about what engagement “should” look like (Haines, 2023; Tervalon and Murray-García, 1998).

### Situating the findings within broader cross-cultural evidence

Our findings resonate with research on Chinese patients in other Western healthcare contexts. Chinese Australian cancer patients reported comparable communication barriers, despite interpreter availability (Hyatt et al., 2017; Levesque et al., 2020), and Chinese Australians found access to desired palliative care blocked by language differences (Lim et al., 2026). A systematic review and meta-synthesis of qualitative studies on shared decision making among Asian Americans identified themes of negotiating power, differing expectations, cultural influences on decision-making, and social support, consistent with our findings (Tan et al., 2022). These parallels across country contexts suggest that the experiences of our participants reflect broader patterns in Chinese migrants' interactions with Western healthcare systems (Gao et al., 2019).

What may be distinctive about our Hong Kong Chinese participants, however, is their historical relationship with British institutions. Having lived under British colonial governance, many expected to feel familiar with UK systems, unlike mainland Chinese or other East Asian migrant groups. The frustration our participants expressed was not simply about encountering an unfamiliar system, but encountering a system they expected to recognize and accommodate them. Poor understanding of these restrictions by healthcare professionals significantly hindered trust-building in patient-clinician relationships (Berry et al., 1987; Finch et al., 2001; Green et al., 2006; Triandis et al., 1988). Our findings underscore the need for UK health and psychosocial care professionals to have easy access to accurate information about visa-related healthcare rights, explaining which should not fall on patients already compromised by cancer (Berry et al., 1987; Finch et al., 2001).

### Pandemic distruptions and community organizations

Beyond cultural and linguistic barriers, participants reported that inconsistency among oncology consultants, unavailability of clinical nurse specialists, and frequent unexplained appointment cancellations contributed to their loss of trust. UK cancer care deteriorated markedly during the pandemic (Cancer Care Research Centre, 2005; Dhada et al., 2021; Lai et al., 2020; Macmillan Cancer Support, 2020; Richards et al., 2020). Half our sample were BNO visa holders with restricted healthcare rights. Chinese community organizations emerged as crucial bridges between participants and UK cancer services, particularly for BNO visa holders with less acculturation (Chan et al., 2007; Liu et al., 2017). They provided linguistically and culturally appropriate support, navigation assistance, and a sense of belonging that was lacking in statutory services.

It is important to note that pandemic-specific systemic disruptions and enduring cultural-linguistic barriers did not operate independently. Rather, pandemic conditions amplified pre-existing cultural barriers. For participants whose cultural orientation already discouraged proactive help-seeking (Theme 3, RQ1) or questioning clinical authority (Theme 1, RQ2), the additional burden of navigating a disrupted system with reduced continuity of care and fewer opportunities for relationship-building with clinicians intensified their sense of disempowerment and mistrust. The frustration about “having to be proactive” reported by participants thus reflects both a culturally unfamiliar expectation within UK healthcare and the heightened demand for patient self-advocacy during a period of service fragmentation. We acknowledge that without a pre-pandemic comparison group, we cannot definitively attribute specific experiences to one factor over the other; however, the cultural ideologies identified in our analysis (RQ2) are well-documented in literature predating the pandemic (e.g., Chan et al., 2007; Warmoth et al., 2017), suggesting that they represent enduring rather than context-specific barriers.

### Implications for clinical practice

Based on our findings and the wider literature, specific strategies could improve UK clinicians' care of Hong Kong Chinese cancer patients.

#### Encouraing expression of concerns

Kleinman's (1980) explanatory model approach provides a practical foundation: brief cultural elicitation questions—such as “What do you think is causing your illness?”, “What do you think your sickness does to you?”, and “How would you like decisions about your treatment to be made?”—can surface misaligned expectations early in the clinical relationship, before they erode trust. A cultural humility stance (Tervalon and Murray-García, 1998) further suggests that these questions should be asked not as a one-off “cultural assessment,” but as an ongoing practice of curiosity and self-reflection throughout the care trajectory. Clinicians can use reframed open-ended prompts that legitimize disclosure while preserving respect, such as by referring to the experience of other patients in the same situation (Sheikh et al., 2008). Question prompt lists (QPLs) in traditional Chinese and framed as tools endorsed by the clinical team can facilitate self-advocacy. When patients decline to ask questions, clinicians should not assume an absence of concerns, but can offer structured summaries, such as by checking their understanding, and inviting correction. These prompts position participation as cooperation with authority rather than as challenge.

#### Explaining and adapting shared decision-making

Research suggests that when shared decision-making is culturally unfamiliar, patients may interpret a clinician's invitation to participate as uncertainty or incompetence (Epner and Baile, 2012; Lee et al., 2022; Obeidat et al., 2013). Explaining the rationale for sharing decisions at the outset, as normal UK practice, and willingness to involve family members, goes some way toward the family-centered decision-making model that is more culturally familiar (Corrigan and Lee, 2020; Qin et al., 2024).

#### Ethical community partnerships

The lack of recruitment from UK mainstream cancer services, with successful recruitment only through a small Chinese community organization, is itself a finding that underscores the limited visibility of Hong Kong Chinese patients within mainstream UK cancer support pathways. Community health workers who share the linguistic and cultural background of the target population can act as intermediaries, providing culturally congruent social support and facilitating access to care (Katigbak et al., 2015; Torres et al., 2014; Taylor et al., 2019). For Hong Kong Chinese cancer patients, bilingual health advocates embedded within existing community organizations could serve as bridges to NHS services. Such partnerships must be structured ethically and equitably, through partnerships between NHS trusts and Chinese community organizations that include: (a) adequate funding and recognition of community organizations' expertise; (b) clear governance arrangements regarding information sharing and patient confidentiality; and (c) co-design of outreach activities concerning the content and mode of delivery. The “invisibility” of the Chinese community to health services emerged as a key barrier to healthcare service access for Chinese migrants in England (Lee et al., 2017).

#### Cultural competency training

A majority of oncology clinicians report uncertainty and discomfort when working with minority patients (Watts et al., 2017). Cultural training should include specific content on East Asian communication norms, the role of family in decision-making, and the potential for stoicism to mask unmet needs (Taylan and Weber, 2023).

### Limitations

The study sample was small (*N* = 10) and predominantly female with breast cancer, the result of an unforeseen curtailing of recruitment. As in other psycho-oncology research, breast cancer patients tend to be active participants (Geller et al., 2011). The study sample limited our ability to draw conclusions about gender-specific or cancer-type-specific patterns. The within-group variations we observed, particularly regarding stoicism, suggest that age, length of residency, and potentially gender interact with cultural ideologies in ways that a small, predominantly female sample cannot fully capture. In addition, the voices of Hong Kong Chinese patients with other cancer types were underrepresented. Patients with rare cancers may face additional psychological challenges arising from low public awareness and difficulty finding specific information and support (Duijts and van der Zwan, 2021; Papadopoulos et al., 2022). As this was a qualitative study, transferability was supported through detailed contextual description, enabling readers to judge relevance to other groups and settings (Lincoln and Guba, 1985).

All participants were recruited through a Chinese community cancer support organization, meaning they had already successfully navigated to community-based support. Our sample may therefore not represent the experiences of the most isolated or marginalized Hong Kong Chinese cancer patients, those who are not connected to any Chinese community organization and who may have disengaged entirely from both mainstream and community support services. The barriers to access and engagement identified in this study should be understood as those experienced by individuals who retained some connection to community support; the experiences of those who have disengaged completely may involve additional or more severe barriers that our study was not positioned to capture.

Consistent with reflexive thematic analysis, the analysis was conducted by the first author, and interpretations were necessarily shaped by her positionality (King and Brooks, 2018). We addressed this through reflexive practices and regular supervisory dialogue (see Methods). Consistent with (Braun and Clarke 2022a,b, 2024), we aimed for transparency about the non-positivist, reflexive approach of this study and its implications for the findings.

### Recommendations for future research

Future studies of a range of cancer types and more male participants could capture broader patterns of experience to examine how gender, age, and acculturation interact with cultural ideologies in shaping engagement with UK cancer services. Longitudinal designs could also help clarify whether the generational differences observed in this study reflect cohort effects, acculturation, or the modernization of attitudes in contemporary Hong Kong. Comparative research with other East and Southeast Asian migrant groups would also help clarify which patterns are shared and which specific to Hong Kong Chinese patients. The positive role of Chinese community organizations warrants further investigation, as limited evidence restricts these organizations' ability to leverage political and funding support (Manchester Oriental Organisations Alliance, 2004). Inclusion of family members and carers in future research would provide valuable perspectives on support needs during and after cancer treatment (Lui et al., 2009; Tang et al., 2007). Since data were collected during the COVID-19 pandemic, and some experiences of service disruption may reflect pandemic-specific conditions rather than routine features of UK cancer care, future research conducted outside pandemic conditions would help clarify which barriers are enduring and which were context-specific. Future research should also explore strategies for reaching Hong Kong Chinese cancer patients who are not connected to Chinese community organizations, including those who may have disengaged from both mainstream and community-based support. This could involve recruitment through NHS cancer services directly, GP practices, or social prescribing link workers, to capture the experiences of the most isolated individuals. Finally, implementation studies should test the practicability and impact of the culturally attuned communication strategies and community partnership models recommended above, and explore UK professionals' perspectives on working with Chinese cancer patients, including their beliefs, assumptions, and training needs.

## Conclusion

As the first study of Hong Kong Chinese cancer patients in the UK, this research demonstrates how culturally shaped expectations around authority, collectivism, and stoicism interact with specific features of UK cancer care to produce communication difficulties, unmet needs, and eroded trust. Practical implications include adaptation of consultation techniques to invite rather than assume patient participation; ethical partnership with Chinese community organizations; and psychosocial pathways that accommodate diverse help-seeking styles. Future research should examine whether such culturally attuned adaptations improve care experiences for Hong Kong Chinese and other East Asian cancer patients in the UK, and incorporate the perspectives of healthcare professionals working with these populations.

## Data Availability

The original contributions presented in the study are included in the article/Supplementary material, further inquiries can be directed to the corresponding author.

## References

[B1] Abraham S. Foreman N. Sidat Z. Sandhu P. Marrone D. Headley C. . (2022). Inequalities in cancer screening, prevention and service engagement between UK ethnic minority groups. Br. J. Nurs. 31, S14–S24. doi: 10.12968/bjon.2022.31.10.S14 35648663

[B2] Anderson C. (2010). Presenting and evaluating qualitative research. Am. J. Pharm. Educ. 74:141. doi: 10.5688/aj7408141 21179252 PMC2987281

[B3] Archer S. Holch P. Armes J. Calman L. Foster C. Gelcich S. . (2020). “No turning back” Psycho-oncology in the time of COVID-19: insights from a survey of UK professionals. Psychooncology 29, 1430–1435. doi: 10.1002/pon.5486 32691451 PMC7404944

[B4] Attride-Stirling J. (2001). Thematic networks: an analytic tool for qualitative research. Qual. Res. 1, 385–405. doi: 10.1177/146879410100100307

[B5] Bedford O. Hwang K. K. (2003). Guilt and shame in Chinese culture: a cross-cultural framework from the perspective of morality and identity. J. Theory Soc. Behav. 33, 127–144. doi: 10.1111/1468-5914.00210

[B6] Berry J. W. Kim U. Minde T. Mok D. (1987). Comparative studies of ccculturative stress. Int. Migr. Rev. 21, 491–511. doi: 10.1177/019791838702100303

[B7] Braun V. Clarke V. (2006). Using thematic analysis in psychology. Qual. Res. Psychol. 3, 77–101. doi: 10.1191/1478088706qp063oa

[B8] Braun V. Clarke V. (2019). Reflecting on reflexive thematic analysis. Qual. Res. Sport Exerc. Health 11, 589–597. doi: 10.1080/2159676X.2019.1628806

[B9] Braun V. Clarke V. (2021). To saturate or not to saturate? Qual. Res. Sport Exerc. Health 13, 201–216. doi: 10.1080/2159676X.2019.1704846

[B10] Braun V. Clarke V. (2022a). Thematic Analysis: A Practical Guide. Los Angeles, CA: Sage. doi: 10.53841/bpsqmip.2022.1.33.46

[B11] Braun V. Clarke V. (2022b). Toward good practice in thematic analysis: avoiding common problems and be(com)ing a knowing researcher. Int. J. Transgend. Health 24, 1–6. doi: 10.1080/26895269.2022.2129597 36713144 PMC9879167

[B12] Braun V. Clarke V. (2024). Supporting best practice in reflexive thematic analysis reporting in Palliative Medicine: a review of published research and introduction to the Reflexive Thematic Analysis Reporting Guidelines (RTARG). Palliat. Med. 38, 289–300. doi: 10.1177/02692163241234800 38469804 PMC11157981

[B13] Cancer Care Research Centre (2005). Summary Report: Patient and Carer Experiences, Public Involvement Study. Stirling: Cancer Care Research Centre, University of Stirling.

[B14] Carreira H. Williams R. Funston G. Stanway S. Bhaskaran K. (2021). Associations between breast cancer survivorship and adverse mental health outcomes: a matched population-based cohort study in the United Kingdom. PLoS Med. 18:e1003504. doi: 10.1371/journal.pmed.1003504 33411711 PMC7822529

[B15] Carter N. Bryant-Lukosius D. DiCenso A. Blythe J. Neville A. J. (2014). The use of triangulation in qualitative research. Oncol. Nurs. Forum 41, 545–547. doi: 10.1188/14.ONF.545-547 25158659

[B16] Chan C. K. Cole C. Bowpitt G. (2007). “Beyond silent organisations”: a reflection of the UK Chinese people and their community organisations. Crit. Soc. Policy 27, 509–533. doi: 10.1177/0261018307081810

[B17] Chau C. M. (2008). Health Experiences of Chinese People in the UK. A Race Equality Foundation Briefing Paper. Available online at: https://raceequalityfoundation.org.uk/health-and-care/health-experiences-of-chinese-people-in-the-uk/ (Accessed May 12, 2023).

[B18] Chau C. M. Yu W. K. (2002). Coping with social exclusion: experiences of Chinese women in three societies. Asian Women 14, 103–127.

[B19] Chau C. M. Yu W. K. (2004). “Pragmatism, globalism and culturalism: health pluralism of Chinese people in Britain,” in The Definition and Construction of Health and Illness: European Perspectives, eds. Shaw I. Kauppinen K. (Aldershot: Ashgate), 65–79.

[B20] Chichirez C. M. Purcărea V. L. (2018). Interpersonal communication in healthcare. J. Med. Life 11, 119–122. 30140317 PMC6101690

[B21] Chien C. L. (2016). Beyond authoritarian personality: the culture-inclusive theory of Chinese authoritarian orientation. Front. Psychol. 7:924. doi: 10.3389/fpsyg.2016.00924 27445894 PMC4927584

[B22] Chu Q. Wong C. C. Y. Chen L. Shin L. J. Chen L. Lu Q. . (2021). Self-stigma and quality of life among Chinese American breast cancer survivors: a serial multiple mediation model. Psychooncology 30, 392–399. doi: 10.1002/pon.5590 33175446 PMC10044476

[B23] Cockle S. Ogden J. (2022). Patients' expectations of cancer treatment and their perceived link to subsequent experiences: a qualitative study. Br. J. Health Psychol. 27, 267–282. doi: 10.1111/bjhp.12544 34173698

[B24] Conway A. Clamp A. Hasan J. Goonetilleke D. Shore K. Wong L. . (2014). Accessing cancer services in North West England: the Chinese population. Eur. J. Cancer Care 23, 345–353. doi: 10.1111/ecc.12171 24393098

[B25] Corrigan P. Lee E.-J. (2020). Family-centered decision making for East Asian adults with mental illness. Psychiatr. Serv. 71, 1219–1221. doi: 10.1176/appi.ps.201900570 33138713

[B26] Creswell J. (2009). Research Design: Qualitative, Quantitative, and Mixed Methods Approaches, 3rd Edn. Los Angeles, CA: Sage.

[B27] Crezee I. H. M. Roat C. E. (2019). Bilingual patient navigator or healthcare interpreter: what's the difference and why does it matter? Cogent Med. 6:181087776. doi: 10.1080/2331205X.2019.1582576

[B28] Denny E. Weckesser A. (2019). Qualitative research: what it is and what it is not. BJOG Res. Methods Guides 126:369. doi: 10.1111/1471-0528.15198 29916201

[B29] Department of Health Food and Health Bureau, and Hospital Authority. (2019). Hong Kong Cancer Strategy 2019. Available online at: https://www.healthbureau.gov.hk/download/press_and_publications/ (Accessed May 12, 2023).

[B30] Dhada S. Stewart D. Cheema E. Hadi M. A. Paudyal V. (2021). Cancer services during the COVID-19 pandemic: systematic review of patient's and caregiver's experiences. Cancer Manag. Res. 13, 5875–5887. doi: 10.2147/CMAR.S318115 34349561 PMC8328387

[B31] Donaldson K. (2021, January 29). UK expects 300,000 people to leave Hong Kong, move to Britain. Bloomberg. Available online at: https://www.bloomberg.com/news/articles/2021-01-29/u-k-expects-300-000-people-to-leave-hong-kong-move-to-britain#xj4y7vzkg (Accessed May 12, 2023).

[B32] Duijts S. F. A. van der Zwan J. M. (2021). Rare cancers and cancer of unknown primary: here's what you should know. Eur. J. Cancer Care 30:e13508. doi: 10.1111/ecc.13508 34755410

[B33] Eberly L. A. Kallan M. J. Julien H. M. Haynes N. Khatana S. A. M. Nathan A. S. . (2020). Patient characteristics associated with telemedicine access for primary and specialty ambulatory care during the COVID-19 pandemic. JAMA Netw. Open 3:e2031640. doi: 10.1001/jamanetworkopen.2020.31640 33372974 PMC7772717

[B34] Edwards P. O'Mahoney J. Vincent S. (eds.). (2014). Studying Organization Using Critical Realism. London: Oxford University Press. doi: 10.1093/acprof:oso/9780199665525.001.0001

[B35] Elkan R. Avis M. Cox K. Wilson E. Patel S. Miller S. . (2007). The reported views and experiences of cancer service users from minority ethnic groups: a critical review of the literature. Eur. J. Cancer Care 16, 109–121. doi: 10.1111/j.1365-2354.2006.00726.x 17371419

[B36] Ellis L. M. Blanke C. D. Roach N. (2005). Losing the battle with cancer. JAMA Oncol. 1, 13–14. doi: 10.1001/jamaoncol.2014.188 26182295

[B37] El-Shakankery K. H. Kefas J. Crusz S. M. (2020). Caring for our cancer patients in the wake of COVID-19. Br. J. Cancer 123, 3–4. doi: 10.1038/s41416-020-0843-5 32303717 PMC7164408

[B38] Epner D. E. Baile W. F. (2012). Patient-centered care: the key to cultural competence. Ann. Oncol. 23, 33–42. doi: 10.1093/annonc/mds086 22628414

[B39] Finch B. K. Hummer R. A. Kol B. Vega W. A. (2001). The role of discrimination and acculturative stress in the physical health of Mexican-origin adults. Hisp. J. Behav. Sci. 23, 399–429. doi: 10.1177/0739986301234004

[B40] Forero R. Nahidi S. De Costa J. Mohsin M. Fitzgerald G. Gibson S. . (2018). Application of four-dimension criteria to assess rigour of qualitative research in emergency medicine. BMC Health Serv. Res. 18:120. doi: 10.1186/s12913-018-2915-2 29454350 PMC5816375

[B41] Fryer T. (2022). A critical realist approach to thematic analysis: producing causal explanations. J. Crit. Realism 21, 365–384. doi: 10.1080/14767430.2022.2076776

[B42] Gale N. K. Heath G. Cameron E. Rashid S. Redwood S. (2013). Using the framework method for the analysis of qualitative data in multi-disciplinary health research. BMC Med. Res. Methodol. 13:117. doi: 10.1186/1471-2288-13-117 24047204 PMC3848812

[B43] Gao S. Corrigan P. Qin S. Nieweglowski K. (2019). Comparing Chinese and European American mental health decision making. J. Ment. Health 28, 59–66. doi: 10.1080/09638237.2017.1417543 29260922

[B44] Geller B. M. Mace J. Vacek P. Johnson A. Lamer C. Cranmer D. . (2011). Are cancer survivors willing to participate in research? J. Community Health 36, 772–778. doi: 10.1007/s10900-011-9374-6 21311959

[B45] Gervais M. C. Jovchelovitch S. (1998). The Health Beliefs of the Chinese Community in England: A Qualitative Research Study. London: Health Education Authority. Available online at: http://eprints.lse.ac.uk/2672/ (Accessed May 12, 2023).

[B46] Given L. M. (2008). The SAGE Encyclopedia of Qualitative Research Methods. Thousand Oaks, CA: SAGE. doi: 10.4135/9781412963909

[B47] Gonzalez P. Lim J. W. Wang-Letzkus M. Flores K. F. Allen K. M. Castañeda S. F. . (2015). Breast cancer cause beliefs: Chinese, Korean, and Mexican American breast cancer survivors. West. J. Nurs. Res. 37, 1081–1099. doi: 10.1177/0193945914541518 25001237 PMC4286528

[B48] Green G. Bradby H. Chan A. Lee M. (2006). “We are not completely Westernised”: dual medical systems and pathways to health care among Chinese migrant women in England. Soc. Sci. Med. 62, 1498–1509. doi: 10.1016/j.socscimed.2005.08.014 16203074

[B49] Haines E. K. (2023). Cancer patients' and survivors' engagement with psychosocial healthcare during the COVID-19 pandemic (Unpublished doctoral dissertation). University College London, London, United Kingdom.

[B50] Haleem A. Javaid M. Singh R. P. Suman R. (2021). Telemedicine for healthcare: capabilities, features, barriers, and applications. Sens. Int. 2:100117. doi: 10.1016/j.sintl.2021.100117 34806053 PMC8590973

[B51] Hanna T. P. King W. D. Thibodeau S. Jalink M. Paulin G. A. Harvey-Jones E. . (2020). Mortality due to cancer treatment delay: systematic review and meta-analysis. BMJ 371:m4087. doi: 10.1136/bmj.m4087 33148535 PMC7610021

[B52] Hawley S. T. Morris A. M. (2017). Cultural challenges to engaging patients in shared decision making. Patient Educ. Couns. 100, 18–24. doi: 10.1016/j.pec.2016.07.008 27461943 PMC5164843

[B53] Healthcare Commission (2008). Report on Self-Reported Experiences of Patients from Black and Minority Ethnic Groups. London: Department of Health.

[B54] Hesse-Biber S. N. (2016). The Practice of Qualitative Research, 3rd Edn. Boston College: Sage.

[B55] Holden C. E. Wheelwright S. Harle A. Wagland R. (2021). The role of health literacy in cancer care: a mixed studies systematic review. PLoS ONE 16:e0259815. doi: 10.1371/journal.pone.0259815 34767562 PMC8589210

[B56] Hou W. K. Lam W. W. Fielding R. (2009). Adaptation process and psychosocial resources of Chinese colorectal cancer patients undergoing adjuvant treatment: a qualitative analysis. Psychooncology 18, 936–944. doi: 10.1002/pon.1457 19090497

[B57] Huang X. Butow P. Meiser B. Goldstein D. (2008). Attitudes and information needs of Chinese migrant cancer patients and their relatives. Aust. N. Z. J. Med. 29, 207–213. doi: 10.1111/j.1445-5994.1999.tb00685.x 10342019

[B58] Hyatt A. Lipson-Smith R. Schofield P. Gough K. Sze M. Aldridge L. J. . (2017). Communication challenges experienced by migrants with cancer: a comparison of migrant and English-speaking Australian-born cancer patients. Health Expect. 20, 886–895. doi: 10.1111/hex.12529 28261937 PMC5600245

[B59] Iyer R. Ring A. (2017). Breast cancer survivorship: key issues and priorities of care. Br. J. Gen. Pract. 67, 140–141. doi: 10.3399/bjgp17X689845 28232363 PMC5325655

[B60] Jin M. X. Kim S. Y. Miller L. J. Behari G. Correa R. (2020). Telemedicine: current impact on the future. Cureus 12:e9891. doi: 10.7759/cureus.9891 32968557 PMC7502422

[B61] Jones D. Neal R. D. Duffy S. R. G. Scott S. E. Whitaker K. L. Brain K. . (2020). Impact of the COVID-19 pandemic on the symptomatic diagnosis of cancer: the view from primary care. Lancet Oncol. 21, 748–750. doi: 10.1016/S1470-2045(20)30242-4 32359404 PMC7251992

[B62] Jovchelovitch S. Gervais M. C. (1999). Social representations of health and illness: the case of the Chinese community in England. J. Community Appl. Soc. Psychol. 9, 247–260. doi: 10.1002/(SICI)1099-1298(199907/08)9:4<247::AID-CASP500>3.0.CO;2-E

[B63] Kagawa-Singer M. Dadia A. V. Surbone A. (2010). Cancer, culture, and health disparities: time to chart a new course? CA Cancer J. Clin. 60, 12–39. doi: 10.3322/caac.20051 20097836

[B64] Karbani G. Lim J. N. Hewison J. Atkin K. Horgan K. Lansdown M. . (2011). Culture, attitude and knowledge about breast cancer and preventive measures: a qualitative study of South Asian breast cancer patients in the UK. Asian Pac. J. Cancer Prev. 12, 1619–1626. 22126509

[B65] Katigbak C. Van Devanter N. Islam N. Trinh-Shevrin C. (2015). Partners in health: a conceptual framework for the role of community health workers in facilitating patients' adoption of healthy behaviors. Am. J. Public Health 105, 872–880. doi: 10.2105/AJPH.2014.302411 25790405 PMC4386525

[B66] King N. Brooks J. M. (2018). “Thematic analysis in organisational research,” in The SAGE Handbook of Qualitative Business Management Research Methods: Methods and Challenges, eds. Cassell C. Cunliffe A. L. Grandy G. (Newcastle upon Tyne: Sage), 219–236. doi: 10.4135/9781526430236.n14

[B67] Kleinman A. (1980). Patients and Healers in the Context of Culture: An Exploration of the Borderland Between Anthropology, Medicine, and Psychiatry. Berkeley, CA: University of California Press. doi: 10.1525/9780520340848

[B68] Koçan S. Gürsoy A. (2016). Body image of women with breast cancer after mastectomy: a qualitative research. J. Breast Health 12, 145–150. doi: 10.5152/tjbh.2016.2913 28331752 PMC5351438

[B69] Kwok C. White K. (2011). Cultural and linguistic isolation: the breast cancer experience of Chinese-Australian women – a qualitative study. Contemp. Nurse 31, 85–94. doi: 10.5172/conu.2011.39.1.85 21955269

[B70] Lai A. G. Pasea L. Banerjee A. Hall G. Denaxas S. Chang W. H. . (2020). Estimated impact of the COVID-19 pandemic on cancer services and excess 1-year mortality in people with cancer and multimorbidity: near real-time data on cancer care, cancer deaths and a population-based cohort study. BMJ Open 10:e043828. doi: 10.1136/bmjopen-2020-043828 33203640 PMC7674020

[B71] Lam J. (2002). Perceptions Against Needs: The Chinese Community in Britain. Presentation to the Liberal Democratic Ethnic Minority Conference. Available online at: https://www.ccrag.org/pages/en/report/libdem-presentation.html (Accessed May 12, 2023).

[B72] Lam W. W. Fielding R. (2003). The evolving experience of illness for Chinese women with breast cancer: a qualitative study. Psychooncology 12, 127–140. doi: 10.1002/pon.621 12619145

[B73] Lawless B. Chen Y. W. (2019). Developing a method of critical thematic analysis for qualitative communication inquiry. Howard J. Commun. 30, 92–106. doi: 10.1080/10646175.2018.1439423

[B74] Lee A. Vedio A. Liu E. Z. H. Horsley J. Jesurasa A. Salway S. . (2017). Determinants of uptake of hepatitis B testing and healthcare access by migrant Chinese in the England: a qualitative study. BMC Public Health 17:747. doi: 10.1186/s12889-017-4796-4 28950835 PMC5615445

[B75] Lee E. W. J. Shi J. (2022). Examining the roles of fatalism, stigma, and risk perception on cancer information seeking and avoidance among Chinese adults in Hong Kong. J. Psychosoc. Oncol. 40, 425–440. doi: 10.1080/07347332.2021.1957061 34357854

[B76] Lee P. Cheong A. Ghazali S. Rashid A. Ong S. C. Ong S. Y. . (2022). Barriers of and strategies for shared decision-making implementation in the care of metastatic breast cancer: a qualitative study among patients and healthcare professionals in an Asian country. Health Expect. 25, 3014–3027. doi: 10.1111/hex.13590 36098241 PMC9700188

[B77] Lee R. M. Vu A. Lau A. (2013). “Culture and evidence-based prevention programs,” in Handbook of Multicultural Mental Health: Assessment and Treatment of Diverse Populations, eds. Paniagua F. Yamada A. M. (San Diego, CA: Elsevier), 121–140. doi: 10.1016/B978-0-12-394420-7.00027-8

[B78] Lee S. Chen L. Ma G. X. Fang C. Y. Oh Y. Scully L. . (2013). Challenges and needs of Chinese and Korean American breast cancer survivors: in-depth interviews. N. Am. J. Med. Sci. 6, 1–8. doi: 10.7156/najms.2013.0601001 24019995 PMC3766352

[B79] Leung L. (2015). Validity, reliability, and generalisability in qualitative research. J. Fam. Med. Prim. Care 4, 324–327. doi: 10.4103/2249-4863.161306 PMC4535087 26288766

[B80] Levant R. F. Wu T. F. Fischer J. (2003). Masculinity ideology: a comparison between U.S. and Chinese young men and women. J. Gend. Cult. Health 1, 137–146. doi: 10.1037/1524-9220.4.1.26

[B81] Levesque J. V. Gerges M. Girgis A. (2019). Psychosocial experiences, challenges, and coping strategies of Chinese-Australian women with breast cancer. Asia Pac. J. Oncol. Nurs. 7, 141–150. doi: 10.4103/apjon.apjon_53_19 32478131 PMC7233569

[B82] Levesque J. V. Gerges M. Wu V. Girgis A. (2020). Chinese-Australian women with breast cancer call for culturally appropriate information and improved communication with health professionals. Cancer Rep. 3:e1218. doi: 10.1002/cnr2.1218 32671993 PMC7941523

[B83] Licqurish S. Phillipson L. Chiang P. Walker J. Walter F. Emery J. (2016). Cancer beliefs in ethnic minority populations: a review and meta-synthesis of qualitative studies. Eur. J. Cancer Care. 26:e12556. doi: 10.1111/ecc.12556 27515153

[B84] Lim C. E. D. Sanchez C. Chen H. (2026). Light in the darkness—accessibility to palliative care for cancer patients of Chinese background and their families. J. Prim. Health Care 16, 373–380. doi: 10.1071/HC24112 41134009

[B85] Lincoln Y. S. Guba E. G. (1985). Naturalistic Inquiry. Newcastle upon Tyne: Sage. doi: 10.1016/0147-1767(85)90062-8

[B86] Linehan M. M. (1993). Cognitive-Behavioural Treatment of Borderline Personality Disorder. New York, NY: Guilford Press.

[B87] Liu S. Qiu G. Louie W. (2017). Use of mindfulness sitting meditation in Chinese American women in treatment of cancer. Integr. Cancer Ther. 16, 110–117. doi: 10.1177/1534735416649661 27252075 PMC5736067

[B88] Lui C. W. Ip D. Chui W. H. (2009). Ethnic experience of cancer: a qualitative study of Chinese-Australians in Brisbane, Queensland. Soc. Work Health Care 48, 14–37. doi: 10.1080/00981380802440403 19197764

[B89] Macmillan Cancer Support (2020). The Forgotten “C”? The Impact of COVID-19 on Cancer Care. Macmillan Cancer Support. Available online at: https://www.macmillan.org.uk/dfsmedia/1a6f23537f7f4519bb0cf14c45b2a629/9601-10061/the-forgotten-c-the-impact-of-covid-on-cancer-care (Accessed May 12, 2023).

[B90] Magadi J. P. Magadi M. A. (2022). Ethnic inequalities in patient satisfaction with primary health care in England: evidence from recent General Practitioner Patient Surveys (GPPS). PLoS ONE 17:e0270775. doi: 10.1371/journal.pone.0270775 36542601 PMC9770381

[B91] Malterud K. Siersma V. D. Guassora A. D. (2016). Sample size in qualitative interview studies: guided by information power. Qual. Health Res. 26, 1753–1760. doi: 10.1177/1049732315617444 26613970

[B92] Manchester Oriental Organisations Alliance (2004). Needs of the Chinese community in the North West Region. Manchester: Manchester Oriental Organisations Alliance.

[B93] Markus H. R. Kitayama S. (1991). Culture and the self: implications for cognition, emotion, and motivation. Psychol. Rev. 98, 224–253. doi: 10.1037/0033-295X.98.2.224

[B94] Mok E. Lam W. M. Chan L. N. Lau K. P. Ng J. S. Chan K. S. . (2010). The meaning of hope from the perspective of Chinese advanced cancer patients in Hong Kong. Int. J. Palliat. Nurs. 16, 298–305. doi: 10.12968/ijpn.2010.16.6.48836 20925293

[B95] Mok M. C. L. Schwannauer M. Chan S. W. Y. (2020). Soothe ourselves in times of need: a qualitative exploration of how the feeling of ‘soothe' is understood and experienced in everyday life. Psychol. Psychother. Theory Res. Pract. 93, 587–620. doi: 10.1111/papt.12245 31369214

[B96] National Children's Centre (1982). The silent minority. The Report of a National Conference on Chinese Families in Great Britain, The Commonwealth Institute, London, United Kingdom.

[B97] Ng C. (2023). Hong Kong must spend more wisely on healthcare, starting with how we pay public doctors. South China Morning Post. Available online at: https://www.scmp.com/comment/opinion/article/3217739/hong-kong-must-spend-more-wisely-healthcare-starting-how-we-pay-public-doctors (Accessed February 12, 2026).

[B98] NICE. (2021). Shared Decision Making. Available online at: https://www.nice.org.uk/guidance/ng197/resources/shared-decision-making-pdf-66142087186885 (Accessed May 12, 2023).

[B99] Obeidat R. F. Homish G. Lally R. (2013). Shared decision making among individuals with cancer in non-Western cultures: a literature review. Oncol. Nurs. Forum 40, 454–463. doi: 10.1188/13.ONF.454-463 23989019

[B100] Office for Health Improvement and Disparities (2023). Mortality Profile Commentary: March 2023. Available online at: https://www.gov.uk/government/statistics/mortality-profile-march-2023/mortality-profile-commentary-march-2023 (Accessed February 12, 2026).

[B101] Office for National Statistics (2021). Census 2021 Results. Available online at: https://census.gov.uk/census-2021-results (Accessed May 12, 2023).

[B102] Office for National Statistics (2022). Long-Term International Migration, Provisional: Year Ending June 2022. Available online at: https://www.ons.gov.uk/peoplepopulationandcommunity/populationandmigration/internationalmigration/bulletins/longterminternationalmigrationprovisional/yearendingjune2022#:~:text=Net%20international%20migration%2C%20which%20is,YE%20June%202021%20(173%2C000) (Accessed May 12, 2023).

[B103] Papadopoulos I. Guo F. Lees S. Ridge M. (2007). An exploration of the meanings and experiences of cancer of Chinese people living and working in London. Eur. J. Cancer Care 16, 424–432. doi: 10.1111/j.1365-2354.2007.00785.x 17760929

[B104] Papadopoulos L. Georgiou C. L. Kee D. Caldwell R. Bourne A. Haurat J. . (2022). The psychosocial impact of the Australian Rare Cancer Portal on patients with rare cancer. J. Clin. Oncol. 40:e24127. doi: 10.1200/JCO.2022.40.16_suppl.e24127

[B105] Patton M. Q. (1990). Qualitative Evaluation and Research Methods, 2nd Edn. Newcastle upon Tyne: Sage Publications, Inc.

[B106] Pinder R. J. Ferguson J. Møller H. (2016). Minority ethnicity patient satisfaction and experience: results of the national cancer patient experience survey in England. BMJ Open 6:e011938. doi: 10.1136/bmjopen-2016-011938 27354083 PMC4932347

[B107] Qin S. Corrigan P. Lee E.-J. (2024). Family-centered decision making: a culturally responsive collaborative approach among Asians living in the United States. Psychiatr. Rehabil. J. 47, 63–71. doi: 10.1037/prj0000603 38407064

[B108] Quaife S. L. Winstanley K. Robb K. A. Simon A. E. Ramirez A. J. Forbes L. J. . (2015). Socioeconomic inequalities in attitudes towards cancer: an international cancer benchmarking partnership study. Eur. J. Cancer Prev. 24, 253–260. doi: 10.1097/CEJ.0000000000000140 25734238 PMC4372163

[B109] Rahool R. Haider G. Shahid A. Shaikh M. R. Memon P. Pawan B. . (2021). Medical and psychosocial challenges associated with breast cancer survivorship. Cureus 13:e13211. doi: 10.7759/cureus.13211 33717749 PMC7943930

[B110] Raleigh V. Holmes J. (2021). The Health of People From Ethnic Minority Groups in England. The King's Fund. Available online at: https://www.kingsfund.org.uk/publications/health-people-ethnic-minority-groups-england (Accessed May 12, 2023).

[B111] Ramirez A. V. Ojeaga M. Espinoza V. Hensler B. Honrubia V. (2021). Telemedicine in minority and socioeconomically disadvantaged communities amidst COVID-19 pandemic. Otolaryngol. Head Neck Surg. 164, 91–92. doi: 10.1177/0194599820947667 32720844

[B112] Raymond E. Thieblemont C. Alran S. Faivre S. (2020). Impact of the COVID-19 outbreak on the management of patients with cancer. Target. Oncol. 15, 249–259. doi: 10.1007/s11523-020-00721-1 32445083 PMC7243433

[B113] Richards M. Anderson M. Carter P. Ebert B. L. Mossialos E. (2020). The impact of the COVID-19 pandemic on cancer care. Nat. Cancer 1, 565–567. doi: 10.1038/s43018-020-0074-y 35121972 PMC7238956

[B114] Roberts K. Dowell A. Nie J. B. (2019). Attempting rigour and replicability in thematic analysis of qualitative research data; a case study of codebook development. BMC Med. Res. Methodol. 19:66. doi: 10.1186/s12874-019-0707-y 30922220 PMC6437927

[B115] Rocque R. Leanza Y. (2015). A systematic review of patients' experiences in communicating with primary care physicians: intercultural encounters and a balance between vulnerability and integrity. PLoS ONE 10:e0139577. doi: 10.1371/journal.pone.0139577 26440647 PMC4594916

[B116] Rudat K. (1994). Black and Minority Ethnic Groups in England: Health and Lifestyles. London: Health Education Authority.

[B117] Runnymede Trust (1986). The Chinese Community in Britain: The Home Affairs Committee Report in Context. London: Runnymede Trust.

[B118] Seven M. Bagcivan G. Pasalak S. I. Oz G. Aydin Y. Selcukbiricik F. . (2021). Experiences of breast cancer survivors during the COVID-19 pandemic: a qualitative study. Support. Care Cancer 29, 6481–6493. doi: 10.1007/s00520-021-06243-4 33905013 PMC8077852

[B119] Sheikh A. Gatrad A. R. Dhami S. (2008). Consultations for people from minority groups. BMJ 337:a273. doi: 10.1136/bmj.a273 18595929 PMC2443593

[B120] Smith B. McGannon K. R. (2018). Developing rigor in qualitative research. Int. Rev. Sport Exerc. Psychol. 11, 101–121. doi: 10.1080/1750984X.2017.1317357

[B121] So W. K. W. Chan D. N. S. Law B. M. H. Rana T. Wong C. L. (2022). Achieving equitable access to cancer screening services to reduce the cancer burden in the Asia-Pacific region: experience from Hong Kong. Lancet Reg. Health West. Pac. 29:100587. doi: 10.1016/j.lanwpc.2022.100587 36605882 PMC9808425

[B122] Spicer J. Chamberlain C. Papa S. (2020). Provision of cancer care during the COVID-19 pandemic. Nat. Rev. Clin. Oncol. 17, 329–331. doi: 10.1038/s41571-020-0370-6 32296166 PMC7156894

[B123] Squires A. (2009). Methodological challenges in cross-language qualitative research: a research review. Int. J. Nurs. Stud. 46, 277–287. doi: 10.1016/j.ijnurstu.2008.08.006 18789799 PMC2784094

[B124] Tam-Ashing K. Padilla G. Tejero J. Kagawa-Singer M. (2003). Understanding the breast cancer experience of Asian American women. Psychooncology 12, 38–58. doi: 10.1002/pon.632 12548647

[B125] Tan N. Q. P. Maki K. Lopez-Olivo M. Geng Y. Volk R. (2022). Cultural influences on shared decision-making among Asian Americans: a systematic review and meta-synthesis of qualitative studies. Patient Educ. Couns. 105, 3521–3532. doi: 10.1016/j.pec.2022.10.350 36344320 PMC11852001

[B126] Tang S. T. Li C. Y. Liao Y. C. (2007). Factors associated with depressive distress among Taiwanese family caregivers of cancer patients at the end of life. Palliat. Med. 21, 249–257. doi: 10.1177/0269216307077334 17641079

[B127] Taylan C. Weber L. T. (2023). “Don't let me be misunderstood”: communication with patients from a different cultural background. Pediatr. Nephrol. 38, 643–649. doi: 10.1007/s00467-022-05573-7 35930048 PMC9842546

[B128] Taylor S. P. (2018). Critical realism vs social constructionism and social constructivism: application to a social housing research study. Basic Appl. Res. 37, 216–222.

[B129] Taylor B. Mathers J. Parry J. (2019). A conceptual framework for understanding the mechanism of action of community health workers services: the centrality of social support. J. Public Health 41, e43–e50. doi: 10.1093/pubmed/fdx161 29228321

[B130] Tebes J. K. (2005). Community science, philosophy of science, and the practice of research. Am. J. Community Psychol. 35, 213–230. doi: 10.1007/s10464-005-3399-x 15909796

[B131] Temple B. Young A. (2004). Qualitative research and translation dilemmas. Qual. Res. 4, 161–178. doi: 10.1177/1468794104044430

[B132] Terry G. Hayfield N. (2020). “Reflexive thematic analysis,” in Handbook of Qualitative Research in Education, eds. Ward M. Delamont S. (Cheltenham: Edward Elgar), 430–441. doi: 10.4337/9781788977159.00049

[B133] Tervalon M. Murray-García J. (1998). Cultural humility versus cultural competence: a critical distinction in defining physician training outcomes in multicultural education. J. Health Care Poor Underserved 9, 117–125. doi: 10.1353/hpu.2010.0233 10073197

[B134] Torres S. Labonté R. Spitzer D. Andrew C. Amaratunga C. (2014). Improving health equity: the promising role of community health workers in Canada. Healthc. Policy 10, 73–85. doi: 10.12927/hcpol.2014.23983 25410697 PMC4253897

[B135] Tran L. (2006). Health Needs of the Chinee in Shropshire County and Telford and Wrekin: A Report Commissioned by Shropshire County Primary Care Trust. London: Chinese National Healthy Living Centre.

[B136] Triandis H. C. Bontempo R. Villareal M. J. Asai M. Lucca N. (1988). Individualism and collectivism: cross-cultural perspectives on self-ingroup relationships. J. Pers. Soc. Psychol. 54, 323–338. doi: 10.1037/0022-3514.54.2.323

[B137] van Nes F. Abma T. Jonsson H. Deeg D. (2010). Language differences in qualitative research: is meaning lost in translation? Eur. J. Ageing 7, 313–316. doi: 10.1007/s10433-010-0168-y 21212820 PMC2995873

[B138] Wang H. H. X. Wang J. J. (2019). Implications of evidence-based understanding of benefits and risks for cancer prevention strategy. Hong Kong Med. J. 25, 346–348. doi: 10.12809/hkmj198192 31761747

[B139] Wang Y. Du S. (2023). Time to rebuild the doctor-patient relationship in China. Hepatobiliary Surg. Nutr. 12, 235–238. doi: 10.21037/hbsn-23-104 37124676 PMC10129885

[B140] Warmoth K. Cheung B. You J. Yeung N. C. Y. Lu Q. (2017). Exploring the social needs and challenges of Chinese American immigrant breast cancer survivors: a qualitative study using an expressive writing approach. Int. J. Behav. Med. 24, 827–835. doi: 10.1007/s12529-017-9661-4 28585073

[B141] Watts K. Meiser B. Zilliacus E. Kaur R. Taouk M. L. Girgis A. . (2017). Communicating with patients from minority backgrounds: individual challenges experienced by oncology health professionals. Eur. J. Oncol. Nurs. 26, 1–7. doi: 10.1016/j.ejon.2016.12.001 28069155

[B142] Wong-Kim E. Sun A. Merighi J. R. Chow E. A. (2005). Understanding quality-of-life issues in Chinese women with breast cancer: a qualitative investigation. Cancer Control 12, 6–12. doi: 10.1177/1073274805012004S02 16327745

[B143] Wu C. S. Warmoth K. M. Cheung B. Loh A. Young L. Lu Q. . (2019). Successful strategies for engaging Chinese breast cancer survivors in a randomised controlled trial. Transl. Issues Psychol. Sci. 5, 51–61. doi: 10.1037/tps0000171 30923730 PMC6433399

[B144] Yu W. K. (2000). Chinese Older People: A Need for Social Inclusion in Two Communities. Bristol: The Policy Press.

[B145] Zhai H. Lavender C. Li C. Wu H. Gong N. Cheng Y. . (2020). Who decides? Shared decision making among colorectal cancer surgery patients in China. Support. Care Cancer 28, 5177–5183. doi: 10.1007/s00520-020-05391-3 32133543

[B146] Zhu L. Li D. Jiang X. L. Jia Y. Liu Y. Li F. . (2022). Effects of telemedicine interventions on essential hypertension: a protocol for a systematic review and meta-analysis. BMJ Open 12:e060376. doi: 10.1136/bmjopen-2021-060376 36175096 PMC9528584

