# Supplementary material for: Chinese people's experience of cancer in the UK: a reflexive thematic analysis
Source: Front Psychol. 2026 Apr 16;17:1745349. doi: 10.3389/fpsyg.2026.1745349 (PMC13128559; doi:10.3389/fpsyg.2026.1745349)
Supplement: Supplementary file 1 [file Supplementary_file_1.docx]

**Appendices**

**Appendix A. Interview Protocols (English and Chinese versions)**

Cancer and psychosocial health: A qualitative study of the experience of Asian Chinese people living in London during COVID-19

Qualitative Interview Guide

1. **Introduction**

*Brief introductions of the study and the interviewer, as well as the aim and structure of the interview.*

Thank you for taking the time to participate in today’s interview.

The aim of this interview is to find out about how cancer affects people who identify themselves as ethnic Asian Chinese people and live in the UK. In particular, we want to understand how they feel about support in the context of the NHS, community, and their own networks. Everyone’s interviews will be put together so that what you say will not be identifiable as you.

This interview will be recorded using the recording function on Microsoft Teams, which I will begin shortly. To maintain confidentiality and anonymity, any identifiable information about you will be removed when the interview is transcribed. The original recording of this interview (in which your voice can be heard) will be stored securely on UCL’s Data Safe Have system, and then permanently deleted after it has been transcribed.

Do you have any questions about anything before we proceed?

***Note:*** The interviewer prompts listed below are not essential and are only to inform any necessary follow-up questions, should the participant only offer limited information to a question.

1. **Turn on the recording function on Microsoft Teams**

Could you please verbally confirm for the purpose of the recording that you consent to participate in this recorded interview?

1. **Participant details**

The following participant physical health details will be gathered:

- Type of cancer diagnosis
- Month of cancer diagnosis
- Type of cancer treatment received (chemotherapy, surgery, or radiotherapy)
- Beginning and end months of cancer treatment
- Whether they were advised to “shield” during COVID-19

1. **Understanding the cancer diagnosis**

I’d like to start by discussing how the experience of receiving a cancer diagnosis has been for you.

- How did you come to know about your cancer diagnosis? (***Interviewer*** ***prompt ideas:*** *probe how the news were told by professionals*)
- How much did you know about your cancer diagnosis when you first got diagnosed?
- What was your immediate emotional and behavioural response to the diagnosis? *(****Interviewer*** ***prompt ideas:*** *probe how they felt, what they thought about self, family, world, illness, death, longevity, and how they reacted to the news)*
- How is a cancer diagnosis generally perceived in your culture? (***Interviewer*** ***prompt ideas:*** *probe cultural perception of cancer, cultural norms on the individual’s immediate and longer-term responses to a cancer diagnosis, attitude to treatment and ways of preserving wellness or ‘wholeness’*)
- In what way do you think your culture has influenced the way you think about your diagnosis? (***Interviewer*** ***prompt ideas:*** *probe impact of the individual’s cultural perspectives on the lens they took on interpreting the different aspects of life*)

1. **Living with cancer in the UK during COVID-19**

We have so far discussed your experience of receiving and understanding a cancer diagnosis. I wonder if we could talk a bit more about your experience of living with cancer during the COVID-19 pandemic, in particular about how the pandemic has had an impact on your cancer care and on your wellbeing.

- While living with cancer, how would you describe the extent to which your everyday life has changed? In what ways has your life changed since receiving the cancer diagnosis? (***Interviewer*** ***prompt ideas:*** *probe reflection on the changes in the physical, psychological, and social aspects of the interviewee’s everyday life, and their interpretations of these changes*)
- How would you describe your experience of living with cancer during the pandemic has been? (***Interviewer*** ***prompt ideas:*** *probe impact of ‘shielding’, social distancing, increased susceptibility to illness, virtual clinic appointments, cancellations of non-urgent procedures, treatment delay, etc.*)
- Do you know anyone else from a similar cultural background who also had cancer? If so, have you heard of how their experience living with cancer has been? *(****Interviewer*** ***prompt ideas:*** *probe the physical, psychological, and social changes that the interviewee mentioned in above)*
- Have you felt able to discuss these culturally influenced thoughts about how you think about cancer with your healthcare professionals in the UK (e.g., your oncologist, CNS, psychologist)?
  - If you felt able to discuss them, how well did you feel you were understood by your healthcare professionals? If you felt they understood you well, could you describe what made you feel understood by your healthcare professionals?
  - If you did not feel able to discuss them, could you describe a bit about why it was challenging for you to share your culturally influenced thoughts about cancer with them? Was there no time? Did you try but that the professionals did not respond? (***Interviewer*** ***prompt ideas:*** *probe who initiated that conversation, professionals’ use of language, manner, tone of voice, clinical setting and their impact on the patient when receiving the information of diagnosis, other facilitators and barriers*)

1. **Wellbeing**

In the next part, we will discuss the role of maintaining good wellbeing when living with cancer during COVID-19.

- In your opinion, how important do you think social and wellbeing support is to ethnic Chinese cancer patients in the UK?
- Are you aware of any services that provide psychological or counselling support for cancer patients in the UK?
  - If yes, which ones are you aware of? Have you ever accessed and engaged with them before?
    - If yes, how did you find the experience? (***Interviewer*** ***prompt ideas:*** *probe perceived barriers/ enablers to accessing and engaging with the support, perceived satisfaction, what was helpful?*)
    - If no, could you share a bit about why you chose to not access the support?
  - If no, what are your views on receiving psychological or counselling support specifically for cancer patients? Would you be willing to learn more about that? Why/ why not?
- Are you aware of any support from community organisations specifically for ethnic Asian Chinese cancer patients in the UK?
  - If yes, which ones are you aware of? Have you ever accessed and engaged with them before?
    - If yes, how did you find the experience? (***Interviewer*** ***prompt ideas:*** *probe perceived barriers/ enabler to accessing and engaging with the programmes, perceived satisfaction, what was helpful?*)
    - If no, could you share a bit about why you chose to not access the support?
  - If no, would you be willing to learn more about support in the community that is specifically for ethnic Asian Chinese cancer patients and their families?
- Other than psychological and counselling support, what else would you like to see in terms of support in the community for ethnic Chinese cancer patients? (***Interviewer*** ***prompt ideas:*** *probe coping with anxiety and low mood, fear of recurrence, return to work, body image, disclosure to future partners or other individuals, f2f or online? Individual or group? etc.)*
- I am aware that family and friends often play a crucial role in Asian Chinese culture. How would you describe the role of your family and friends in your cancer journey? (***Interviewer*** ***prompt ideas:*** *probe how helpful/ unhelpful?*)
- What made it easy or difficult for you to talk about your cancer experience with your family/ friends? *(****Interviewer*** ***prompt ideas:*** *probe stoicism, common cultural beliefs about shame, having to keep the illness secret and private within the family)*

We have now come to the end of our interview today. Thank you so much for coming and for genuinely sharing your experience and thoughts with me. Please do not hesitate to contact the research team should you have any questions.

**Turn off the recording.**

**癌症與精神健康：探討倫敦華人在新冠肺炎疫情下的患癌經歷和心理需要**

**採訪指南**

**介紹**

採訪者會首先向志願參加者簡單介紹這次研究的主題、採訪目的以及採訪過程：

「這項研究希望能透過訪問居住在英國倫敦的華人癌症患者，深入了解倫敦華人在新冠肺炎疫情下的患癌的經歷，以及探討倫敦華人在患癌期間的精神健康和心理需要。

這次研究已經通過倫敦大學學院的倫理審批程序，並已獲得批准招募志願者參與今天的採訪。

在介紹完畢之後，採訪過程將會透過 Microsoft Teams內置的功能開始進行錄音和錄影，直至採訪結束為止。訪問中錄製的影像和音訊會在訪問結束後即時被儲存在倫敦大學學院的 Data Safe Haven 系統，以保障資料的保密和安全。訪問中錄音和錄影的內容會被抄錄（Transcription）並翻譯（Translation）成為全英文的文字檔案，以作研究分析的用途。在抄錄的過程中，研究人員（即本人）會將錄像所得的內容和資訊以匿名的方式進行處理，並且移除所有受訪者的個人可識別資訊。在抄錄及翻譯的過程結束之後，研究人員將會永久刪除在訪問中所得的視訊檔案，並且不會將受訪者的影像和音訊保留任何備份。這次研究已經通過倫敦大學學院資料保護規範的審批程序（註冊編號：Z6364106/2022/08/05 social research）。

研究分析的結果將會成為本人撰寫倫敦大學學院臨床心理學博士論文的原材料。因此，本人衷心感謝受訪者以志願性質參與今次的訪問。

在訪問及錄影開始之前，請問你對上述資料有沒有什麼問題？」

**［開始錄影］**

請受訪者再次以口述的方式確認對授權予採訪者進行是次訪問的錄影。

**採訪開始**

「第一部分，請回答以下有關個人資料的問題。」

- 你患上了什麼癌症？
- 你是在那個月份確診患上癌症？
- 你曾經接受過那種癌症治療？
- 你是在那個月份開始及結束你的癌症治療？
- 你在新冠肺炎疫情期間有否被建議 “shielding”？

「第二部分，我們將探討你在確診患上癌症時的過程和感受。」

- 你是如何得知你患上了癌症？
- 當時的你，面對被確診的癌症有什麼程度的認識？（引導被訪者思考其與主診醫生初次見面時的交流內容和方式的恰當性）
- 當時的你，面對確診癌症時有什麼即時的情緒或反應？（引導被訪者回想並思考其當時在得知確診罹患癌症時的情感反應，行為，以及對於自我、家人、和世界觀的各種想法，例如健康、疾病、壽命等等）
- 在華人文化社會之中，你認為社會大眾對癌症有什麼看法或標籤？（引導被訪者思考華人社會大眾對於癌症的常見標籤和定義，以及引導被訪者思考癌症對於患者人生的長短期影響，以致大眾對於癌症治療的看法）
- 你認為上述社會大眾對癌症的看法對於你如何看待患癌有影響嗎？如有，請問是那些方面的影響？（引導被訪者思考自身文化和社會背景對於自己自待事情時有何影響）

「第三部分，我們將探討你在新冠肺炎的疫症下患癌的經歷和感受。」

- 在患癌期間，你的生活出現了什麼程度的改變？（引導被訪者思考自己在患癌後以及在新冠肺炎的疫症下所有的生理、 心理、以致日常生活中的改變，以及引導被訪者思考自己如何看待這些改變）
- 你會如何描述新冠肺炎的疫情對於你的影響？（引導被訪者思考 “shielding” 、維持社交距離、跟醫院的預約被取消或改成網上或電話形色咨詢對於癌友的影響等等」）
- 你認識其他同是擁有華人文化背景的癌症患者嗎？如有，你聽聞過他／她們的患癌經歷嗎？（引導被訪者思考親友在患癌後所作出的生理、 心理、以致日常生活中的改變，以及引導被訪者思考別人如何看待這些改變）
- 對於患癌後在生活和心理層面上出現的改變，你曾經和負責在英國為你提供癌症治療的醫生和護士表達你的感受和需要嗎？
  - 如有，你認為為你提供治療的英國醫護人員有用心聆聽你的需要和明白你的感受嗎？
    - 如有，他們是怎樣讓你感覺到他們曾用心聆聽你的需要和明白你的感受？
  - 如沒有，你認為是什麼令你感到不太願意和你在英國的醫療團隊分享你對於癌症的想法和感受？（引導被訪者思考是否和醫生交談時時間的充裕程度，醫生在交談時的態度，環境因素的影響等等）

「第四部分，我們將探討在新冠肺炎的疫症下患癌期間維持精神健康的重要性。」

- 你認為在新冠肺炎的疫症下患癌期間維持精神健康重要嗎？為什麼？
- 你知道在英國坊間有什麼途徑可以尋求專門為癌症患者而設的心理治療或心理輔導嗎？（以查看被訪者是否知悉MacMillan, Maggie’s, 及某些 NHS cancer services 提供的心理治療服務）
  - 如你知悉，你曾經使用過這種服務嗎？
  - 如你不知悉，你對於專門為癌症患者而提供的心理治療或心理輔導服務有什麼看法？（引導被訪者思考是什麼因素促使他／她們使用該服務，以及他／她們對於該服務的用後感）
  - 如沒有，你願意分享為什麼你沒有使用該服務嗎？
- 你知道在英國有專門為華人癌症患者提供協助的非牟利機構嗎？
  - 如你知悉，你曾經接受過這些機構所提供的協助嗎？
    - 如有，你曾經接受過什麼協助？
    - 你對於該機構所提供的協助有什麼看法？（引導被訪者思考是什麼因素促使他／她們使用該服務，以及他／她們對於該服務的用後感）
  - 如你不知悉，你認為你會願意了解更多有關機構所提供的協助嗎？
    - 如願意，可否分享一下為什麼你會想接觸或得到什麼類型的協助？
    - 如不願意，可否分享一下你為什麼不願意去了解更多坊間對於華人癌症患者所提供的協助？
- 除了上述所提及到的心理治療和輔導之外，你認為居英的華人癌症患者還有什麼其他方面的需要？（引導被訪者思考以下各項因素：焦慮，抑鬱，重返工作岡位，面對患癌後生理或體形上的改變，以致面對親友時的困難等等）
- 你認為你的家人或摯友在你患癌期間扮演了什麼角色？
- 是什麼令你感到容易／不容易向親友訴說你的患癌經歷和感受？（再次引導被訪者思考華人社會中常見的文化現象，例如「家醜不可外揚」的觀念如何有機會令某些華人癌友在脆弱時也堅持要在外人面前裝堅強，或是寧願婉拒別人提供的協助，也不願將自己患癌的事實告知他人等等）

**［錄影完畢］**

**總結**

「我們已來到採訪的尾聲。衷心感謝你今天的參與和協助。假如你希望得知是次研究項目的結果，請聯絡是次研究的負責人莫紫寧」

**Appendix B. COREQ Checklist**

**Supplementary material**

**COREQ checklist**

| ***Item*** | ***Guide questions/description*** | ***Reported on page*** |
| --- | --- | --- |
| ***Domain 1: Research team & reflexivity*** | | |
| ***Personal characteristics*** | | |
| 1. Interviewer/ facilitator | Which author/s conducted the interview or focus group? | First author: p 6 |
| 2. Credentials | What were the researcher’s credentials? E.g. PhD, MD | pp 7 – 8 |
| 3. Occupation | What was their occupation at the time of the study? | pp 7 – 8 |
| 4. Gender | Was the researcher male or female? | pp 7 – 8 |
| 5. Experience & training | What experience or training did the researcher have? | pp 7 – 8 |
| ***Relationship with participants*** | | |
| 6. Relationship established | Was a relationship established prior to study commencement? | No relationship beyond recruitment correspondence: p 6 |
| 7. Participant knowledge of the interviewer | What did the participants know about the researcher? e.g. personal goals, reasons for doing the research | Participants had no information about researcher other than aims of research in participant information: p 6 |
| 8. Interviewer characteristics | What characteristics were reported about the interviewer/ facilitator? e.g. Bias, assumptions, reasons and interests in the research topic | Hong Kong Chinese trainee clinical psychologist with experience working in a cancer service interviewing other Hong Kong Chinese cancer patients, committed to improving the healthcare experience of immigrants and ethnic minority in the UK: p 7 – 8 |
| ***Domain 2: Study design*** | | |
| ***Theoretical framework*** | | |
| 9. Methodological orientation and Theory | What methodological orientation was stated to underpin the study? e.g. grounded theory, discourse analysis, ethnography, phenomenology, content analysis | pp 5 – 8 |
| ***Participant selection*** | | |
| 10. Sampling | How were participants selected? e.g. purposive, convenience, consecutive, snowball | Volunteers from advertisement on Chinese Association for Cancer Care WhatsApp support group: p 8 |
| 11. Method of approach | How were participants approached? e.g. face-to-face, telephone, mail, email | Email and telephone correspondence with potential participants: p 6 |
| 12. Sample size | How many participants were in the study? | 11: p 6 |
| 13. Non-participation | How many people refused to participate or dropped out? Reasons? | One drop-out due to departure from the UK: p 6 |
| ***Setting*** | | |
| 14. Setting of data collection | Where was the data collected? e.g. home, clinic, workplace | Online, participants at home: p 9 |
| 15. Presence of non-participants | Was anyone else present besides the participants and researchers? | No: p 9 |
| 16. Description of sample | What are the important characteristics of the sample? e.g. demographic data, date | 8 women and 2 men, mixed ages, all identified as Hong Kong Chinese, marital status, no. of children, employment status, years of living in the UK, visa status, primary cancer diagnosis, years of cancer diagnosis. See Table 1. |
| ***Data collection*** | | |
| 17. Interview guide | Were questions, prompts, guides provided by the authors? Was it pilot tested? | Guide created and revised by the first author with two psycho-oncology colleagues in the NHS and reviewed by an Expert by Experience: p 6  Pilot by role play. Pilot on first participant generated no changes. |
| 18. Repeat interviews | Were repeat inter views carried out? If yes, how many? | No repeat interviews |
| 19. Audio/visual recording | Did the research use audio or visual recording to collect the data? | Video recording of interviews kept for transcription and data analysis and then deleted upon completion of transcribing: p 6 |
| 20. Field notes | Were ﬁeld notes made during and/or after the interview or focus group? | Brief notes taken before and after interviews: p 6 |
| 21. Duration | What was the duration of the interviews or focus group? | Approximately one hour: p 6 |
| 22. Data saturation | Was data saturation discussed? | pp 7 – 8 |
| 23. Transcripts returned | Were transcripts returned to participants for comment and/or correction? | No |
| **Domain 3: Analysis and ﬁndings** | | |
| ***Data analysis*** | | |
| 24. Number of data coders | How many data coders coded the data? | One: p 7 – 8 |
| 25. Description of the coding tree | Did authors provide a description of the coding tree? | No |
| 26. Derivation of themes | Were themes identiﬁed in advance or derived from the data? | Derived from data: p 7 |
| 27. Software | What software, if applicable, was used to manage the data? | NVivo: p 7 |
| 28. Participant checking | Did participants provide feedback on the ﬁndings? | Participants did not provide feedback on the findings. |
| ***Reporting*** | | |
| 29. Quotations presented | Were participant quotations presented to illustrate the themes/ﬁndings? Was each quotation identiﬁed? e.g. participant number | Yes: pp 11 – 26 |
| 30. Data and ﬁndings consistent | Was there consistency between the data presented and the ﬁndings? | Yes: Results pp 11 – 26, Discussion: pp 27 – 35 |
| 31. Clarity of major themes | Were major themes clearly presented in the ﬁndings? | Yes, in Results: pp 11 – 26, and in thematic maps: Fig 1 and 2 |
| 32. Clarity of minor themes | Is there a description of diverse cases or discussion of minor themes? | Yes: in Results pp 11 – 26 |
